# Exploring the effects of probiotics on olanzapine-induced metabolic syndrome through the gut microbiota

**DOI:** 10.1186/s13099-024-00664-2

**Published:** 2024-12-21

**Authors:** Syed Mushraf, Kiran Chawla, Shaik Mohammed Abdul Fayaz, Aranjani Jesil Mathew, Gayam Prasanna Kumar Reddy, Mohandas Rao Kappettu Gadahad, Padmaja A. Shenoy, Vasudha Devi, Shalini Adiga, Veena Nayak

**Affiliations:** 1 Division of Pharmacology, Department of Basic Medical Sciences, Manipal Academy of Higher Education, Manipal, Karnataka 576104 India; 2 Department of Microbiology, Kasturba Medical College, Manipal, Manipal Academy of Higher Education, Manipal, Karnataka 576104 India; 3 Department of Biotechnology, Manipal Institute of Technology, Manipal Academy of Higher Education, Manipal, Karnataka 576104 India; 4 Department of Pharmaceutical Biotechnology, Manipal College of Pharmaceutical Sciences, Manipal Academy of Higher Education, Manipal, Karnataka 576104 India; 5 Division of Anatomy, Department of Basic Medical Sciences, Manipal Academy of Higher Education, Manipal, Karnataka 576104 India; 6 Department of Pharmacology, Kasturba Medical College, Manipal, Manipal Academy of Higher Education, Manipal, Karnataka 576104 India

**Keywords:** Probiotics, Gut, Microbiota, 16S rRNA, Metagenomic, Metabolic syndrome, Olanzapine, *Firmicutes*, *Bacteroidetes*

## Abstract

**Background:**

Maintaining gut microbial homeostasis is crucial for human health, as imbalances in the gut microbiota (GM) can lead to various diseases, including metabolic syndrome (MS), exacerbated by the use of antipsychotic medications such as olanzapine (OLZ). Understanding the role of the GM in OLZ-induced MS could lead to new therapeutic strategies. This study used metagenomic analysis to explore the impact of OLZ on the GM composition and examined how probiotics can mitigate its adverse effects in a rat model. Changes in weight, blood pressure, and lipid levels, which are key parameters defining MS, were assessed. Additionally, this study investigated serotonin, dopamine, and histopathological changes to explore their possible link with the microbiota-gut-brain axis (MGBA).

**Results:**

OLZ had an antagonistic effect on serotonin and dopamine receptors, and it was consistently found to alter the composition of the GM, with an increase in the relative abundance (RA) of the *Firmicutes*/*Bacteroidetes* phyla ratio and *TM7* genera, indicating that the anticommonsal action of OLZ affects appetite and energy expenditure, contributing to obesity, dyslipidemia and increased blood pressure, which are core components of MS. Hepatic steatosis and intestinal damage in OLZ-treated rat tissues further indicate its role in MS. Conversely, the administration of probiotics, either alone or in combination with OLZ, was found to mitigate these OLZ-induced symptoms of MS by altering the GM composition. These alterations included increases in the abundances of the taxa *Bacteroidetes*, *Actinobacteria*, *Prevotella*, *Blautia*, *Bacteroides*, *Bacteroidales*, and *Ruminococcaceae* and a decrease in Firmicute abundance. These changes helped maintain gut barrier integrity and modulated neurotransmitter levels, suggesting that probiotics can counteract the adverse metabolic effects of OLZ by restoring the GM balance. Moreover, this study highlights the modulation of the MGBA by OLZ as a potential mechanism through which probiotics modulate serotonin and dopamine levels, influencing metabolic health.

**Conclusion:**

These findings emphasise the significant impact of OLZ on the GM and its contribution to MS. These findings suggest that interventions targeting the GM, such as probiotics, could mitigate the metabolic side effects of OLZ. Future research should focus on developing integrative treatment approaches that consider the health of the gut microbiome in managing antipsychotic-induced adverse effects.

**Supplementary Information:**

The online version contains supplementary material available at 10.1186/s13099-024-00664-2.

## Background

Olanzapine (OLZ) acts by competitively blocking D_4_, 5-HT_2A_ and 5-HT_2C_. They are also antagonistic to D_2_ (weak), α_1_, α_2_ M_1_, and H_1_ receptors [1, 2]. These antagonistic effects can lead to weight gain and can also result in dyslipidemia, ultimately leading to metabolic syndrome (MS), which is a troublesome adverse effect of OLZ [1–3]. MS can be diagnosed when any three of the following criteria are met: high waist circumference, triglyceride levels ≥ 150 mg/dl, high-density lipoprotein cholesterol levels < 40–50 mg/dl, mean arterial pressure ≥ 100 mmHg, and fasting blood glucose levels ≥ 100 mg/dl [4]. Approaches to managing and controlling antipsychotic-induced MS include nutritional and physical activity, counselling, switching to an alternate second-generation antipsychotic, and adjunctive pharmacological interventions in line with current clinical guidelines. However, these strategies have drawbacks, such as lack of acceptance and adherence, increased risk of worsening or relapsing illness, need for more frequent follow-up and monitoring, potential lack of efficacy, increased risk of drug interactions and adverse effects, additional cost, and increased risk of noncompliance.

Microorganisms and humans have coevolved and created symbiotic connections [5]. The phrase “microbiota” refers to microbial communities found in a host [6]. The gut microbiota** (**GM) is the collective name for the vast number of microorganisms in the human gastrointestinal tract [7], and the genes carried by these cells make up the human microbiome [6]. The major phyla within the GM include *Firmicutes*, *Bacteroidetes*, *Proteobacteria*, *Actinobacteria*, *Fusobacteria*, and *Verrucomicrobia* [8], which are essential for maintaining physiological homeostasis. Disruptions in this microbial equilibrium, known as dysbiosis, have been linked to various health issues, including obesity and MS [9]. The microbiota-gut-brain axis (MGBA) involves intricate communication systems within the gastrointestinal tract, resident microorganisms, and the peripheral and central nervous systems (CNS). The MGBA involves neurological, endocrine, and immunological pathways, and growing evidence suggests that it may play a significant role in the development of obesity and related metabolic disorders [8, 10].

OLZ-induced MS has recently been linked to an alteration in the gut microbiome in rats and humans [11, 12], potentially leading to metabolic disorders [13]. Considering the pathology of OLZ-induced dysbiosis, the focus of recent research has shifted towards probiotics. Probiotics have been in use since the early twentieth century. Currently, probiotics are widely recognised for their role in promoting a healthy balance of gut bacteria, offering the host many health benefits when consumed in adequate amounts [11]. Probiotics are currently defined as “live organisms that, when administered in sufficient quantities, provide a health benefit to the host” [14]. Oral administration of probiotics has been shown to effectively manipulate the GM, providing a vital tool for combating MS caused by a high-fat diet. Different strains of beneficial microbes, such as *L*. *rhamnosus* [15], *Lacticaseibacillus paracasei*, *Akkermansia muciniphila* [16], *L. acidophilus*, *L*. *casei*, *B*. *animalis* [17], *L. gasseri* [18], and *Bacillus* spp., have been utilised either alone or in combination and have demonstrated encouraging outcomes in addressing metabolic problems such as obesity, insulin resistance, and hepatic steatosis in rodents and humans fed a high-fat diet.

Given the challenges associated with the use of OLZ for the treatment of psychiatric disorders, including its potential to induce weight gain and MS, this study focused on an innovative approach to investigating the role of the GM in OLZ-induced MS development and its potential modulation by probiotics. Specifically, this study sought to explore the modulation of the GM through probiotic supplementation as a potential strategy to mitigate the metabolic side effects of OLZ.

## Methods

### Selection of animals, their care and diet

Wistar albino rats were chosen because of their genetic uniformity, which minimizes experiment variability, well-characterized physiology, similarities to human disease models, and ethical considerations. Two- to three-month old healthy adult male rats bred in house were procured from institutional facilities after institutional animal ethics committee (IAEC) clearance was obtained. Standard animal care and diet were maintained. The use of Wistar rats in psychiatric disease models is supported by their genetic diversity, well-characterized behavioral patterns, neurophysiological relevance, pharmacological sensitivity, ease of handling, and availability of comparative data. This combination makes them a versatile and reliable model for studying psychiatric disorders. Since the present study’s objective was to evaluate probiotics’ effects on olanzapine-induced metabolic syndrome, we chose a rat model. A psychiatric disease model was not chosen since the objective of this study was not to study the effect of probiotics on psychosis [19–21, 24, 25].

### Drugs, reagents, and other materials

OLZ (obtained locally), ELISA kits (Eagle Biosciences), DNA extraction kits, chemical reagents, and other materials were obtained from commercial sources. Institutional instruments were used to analyse the parameters. The probiotic sachet (a freeze–dried preparation containing a mixture of *Streptococcus thermophiles*, *Bifidobacterium longum/lactis*, *Bifidobacterium breve*, *Bifidobacterium infantis*, *Lactobacillus acidophilus*, *Lactobacillus plantarum*, *Lactobacillus paracasei*, *Lactobacillus delbrueckii* subsp*. bulgaricus*) was procured from ACTIAL FARMACEUTICA Srl, Rome. Based on previous studies, this combination of strains effectively mitigated high-fat diet-induced MS by reducing gut microbial diversity [3, 22–24], hence this combination of strains was chosen for the present study.

### Selection of the dose and preparation of the test drug

OLZ tablets were made into a fine powder, mixed with 0.1 ml of glacial acetic acid, and then made to volume using normal saline. The probiotic sachet was dissolved in phosphate-buffered saline [3]. OLZ and probiotic doses were chosen based on previous animal studies [3, 22–24].

### Experimental methods

In the present study, the treatment groups were divided into six groups. Group I [normal control (N)] was orally administered 1 ml/kg/day of normal saline, group II [OLZ (O)] received OLZ at 2 mg/kg/day i.p., and group III [probiotic-I {low dose} (PM)] and IV [probiotic-II {high dose} (PH)] were orally administered 0.6 g/kg/day and 1.2 g/kg/day probiotics, respectively. The fifth and sixth groups were treated with OLZ at 2 mg/kg/day i.p., followed by probiotics administered orally at 0.6 g/kg/day [Group V {OLZ + probiotic-I} (TM)] and 1.2 g/kg/day [Group VI {OLZ + probiotic-II} (TH)], respectively. All the groups were treated for 90 days. Each group consisted of 6 rats, totaling 36 animals for the entire study. Six animals were allocated to each group based on data from numerous previous studies [3, 22, 25].

### Parameters assessed

1. Body weight (BW) assessment: each animal’s BW was checked at baseline and once every 15 days for 3 months.
2. Blood pressure determinations: conscious animals were acclimated for 5 min before positioning the occlusion and volume pressure recording (VPR) cuffs near the base of the tail, connected to the CODA® monitor, were positioned for blood pressure measurements. They were then allowed to thermoregulate for another 5 min, maintaining a temperature of 32 to 35 °C. Blood pressure readings were taken thrice per session using the CODA® NIBP instrument [26] at baseline and biweekly for 3 months. These readings, recorded in mm Hg, were used to calculate the mean arterial pressure (MAP) for analysis.

The formula for MAP was as follows: {diastolic blood pressure + 1/3 (systolic BP − diastolic blood pressure)}.

### Biochemical analysis

#### Blood sample collection for biochemical estimations

Blood was collected from the retro-orbital plexus from the inner canthus of the eye using capillary tubes; the serum was separated using a refrigerated centrifuge at 4000 rpm for 5 min, which was used for biochemical estimations using respective ELISA kits [3]. Biochemical estimations of triglycerides (TGs), total cholesterol (TC), and high-density lipoprotein cholesterol (HDL-C) were carried out according to standard protocols [23]. All values were noted in mg/dl at baseline and biweekly for 3 months [19, 20]. The serum was also analysed using kits for serotonin and dopamine levels [19, 20]. To compare the results, serum serotonin levels were measured at baseline and at the end of the study.

### Histopathological evaluation of intestinal and hepatic tissues

After 90 days, the rats were sacrificed by cervical dislocation, and intestinal and liver tissues were obtained and stained with hematoxylin and eosin (H&E) [25]. Representative photomicrographs of H&E-stained intestines and liver cross-sections were observed under a light microscope (40× and 10× magnifications) [26]. A qualified expert blinded to the group assignment observed and evaluated all the samples.

### 16S rRNA metagenomic analysis of rat faecal samples

#### Methodology: DNA extraction, PCR amplification, and sequencing of rat faecal pellets

The faecal samples were collected at baseline and on day 90 and immediately stored at − 80 °C. The pellets were outsourced for DNA extraction and sequencing. The quality of the quantified DNA was confirmed by the 1% agarose gel procedure, and the results have been added to the supplementary files. The V3–V4 hypervariable regions of the 16S rRNA gene were amplified with specific bacterial primers forward (5′CCTACGGGNGGCWGCAG3′) and reverse (GACTACHVGGGTATCTAATCC3′) by a thermocycler PCR system (GeneAmp 9700, ABI, USA). PCR products were extracted from a 2% (w/v) agarose gel, further purified by using the AxyPrep DNA Gel Extraction Kit (Axygen Biosciences, Union City, CA, USA) and quantified using QuantiFluor-ST (Promega, USA) following the manufacturers’ protocols. The Sequencing was performed using the Illumina MiSeq system using the MiSeq v3 Reagent Kit to generate pair end (2X300 bp) reads.

**Fig. 1:**
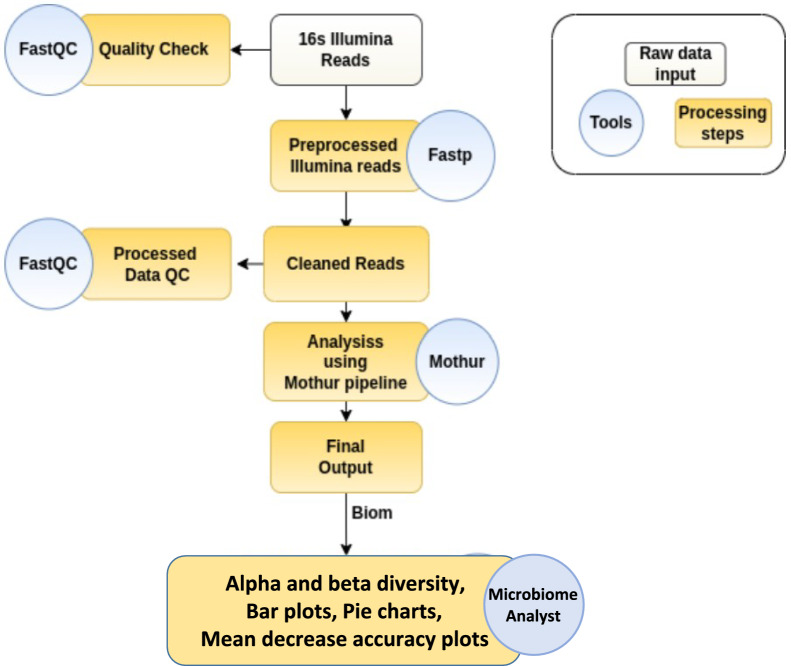
Workflow of 16S rRNA metagenomic analysis of rat faecal samples

The quality of the raw data was assessed using FastQC v.0.11.9 (with the default parameters). The raw fastq reads were preprocessed using Fastp v.0.20.1 [27]. Postfiltering cleaned data were reassessed using FastQC. The Fastq reads were further processed for taxonomic assignment using the Mothur pipeline (Fig. 1) [28]. This includes trimming and cleaning the reads and their taxonomic assignment using the Silva database [29, 30]. The amplicon sequence variant and taxonomy files were further used for downstream analysis. Bar plots and mean decrease accuracy (MDA) plots were generated using MicrobiomeAnalyst [31].

The community richness (Chao1) and diversity within each sample were estimated using the Alpha diversity Shannon and Simpson indices, using the Mothur-based commands. Beta diversity was analyzed using Bray–Curtis metrics and visualized with Principal Coordinate Analysis (PCoA). The permutational multivariate analysis of variance (PERMANOVA) assessed the significance of differences in microbial community between samples from different groups. The alpha and beta diversity plots were generated using MicrobiomeAnalyst [31].

#### Biomarker analysis

The important taxa that might act as biomarkers to differentiate the various groups in this study were identified using MDA plots. The MDA plots are based on the machine learning method random forests (RF), an ensemble learning method for classification, regression, and other tasks. It provides estimates of what variables are important in the classification of data. The MDA plots were analysed, which are a fundamental outcome of the RF forest, and they show how important it is in classifying the data for each variable/taxon. The MDA plot expresses how much accuracy the model loses by excluding each variable. The greater the accuracy suffers, the more influential the variable is for successfully classifying the groups. A higher MDA value indicates the importance of that taxa in predicting or differentiating the groups (Fig. 24A, B) (Supplementary Figures 1 and 2).

#### Statistical analysis

Repeated measures ANOVA and post hoc Bonferroni were applied to compare the continuous variables at different time points across multiple groups. The PERMANOVA test was applied to compare the compositional analysis among the various groups. These analyses were performed using SPSS version 16 software, and a p-value ≤ 0.05 was considered to indicate statistical significance.

## Results

**Fig. 2:**
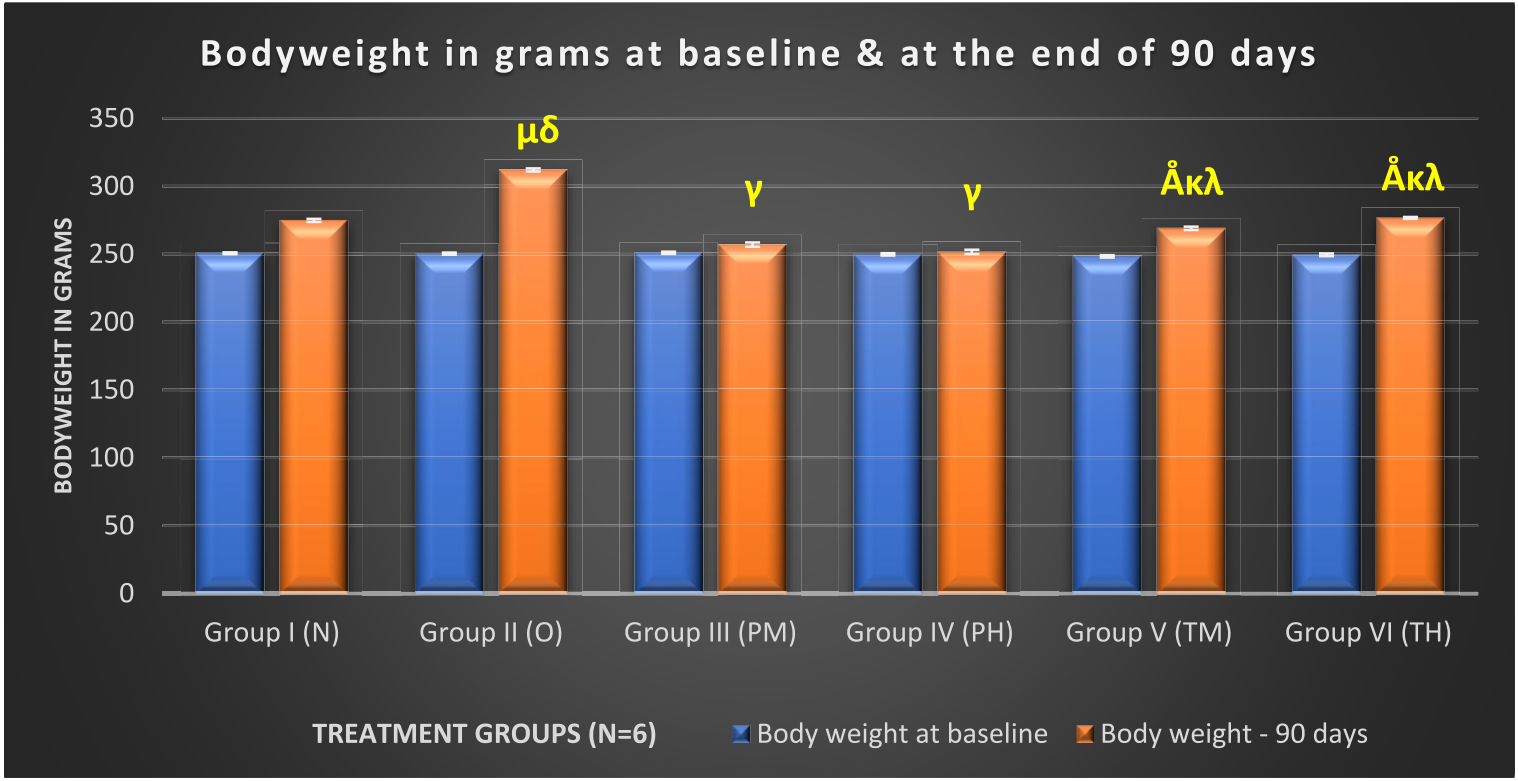
Mean of comparison of BW between various experimental groups at baseline at the end of 90 days. μ: compared to (vs) N (p < 0.001); Å: vs O (p < 0.001); κ: vs PM (p < 0.001); λ: vs PH (p < 0.001); δ: vs groups PM, PH, TM, and TH (p < 0.001), γ: vs. N, O, TM, TH (p < 0.001)

After 15, 30, 45, 60, 75, and 90 days of treatment (Supplementary Table 1), OLZ treatment caused significant weight gain in rats compared to the normal control group (p < 0.001), emphasising the substantial impact of OLZ on BW. The weights of the animals in the low-dose (p < 0.05) and high-dose probiotic (p < 0.001) groups were significantly lower than those in the OLZ and N groups on days 15, 30, and 45, and in comparison, the weights of the test groups (V and VI) (p < 0.001) were significantly lower on days 60, 75, and 90 days. Both groups V and VI demonstrated significant weight control from 15 to 90 days of treatment compared to the OLZ group (p < 0.001) and both probiotic-only treated groups (Fig. 2).

**Fig. 3:**
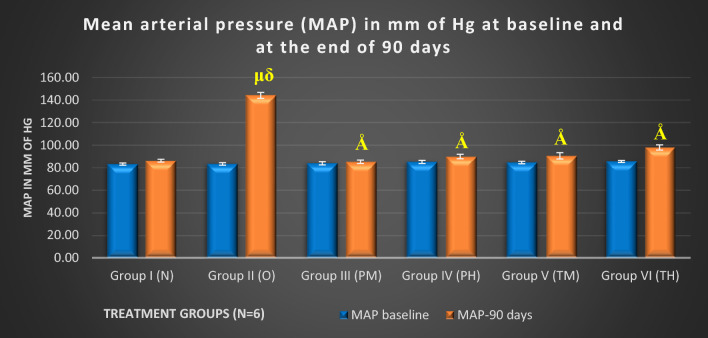
Mean of comparison of MAP between various experimental groups at baseline at the end of 90 days. μ: compared to (vs) N (p < 0.001); δ: vs groups PM, PH, TM, and TH (p < 0.001); Å: vs O (p < 0.001)

After 15 days, OLZ treatment significantly increased the MAP of the rats compared to that in the N, PM, and PH groups (p < 0.001). After 30 days, the MAP was lower in the PM (p < 0.05), PH (p < 0.001), and TM (p < 0.05) groups than in the OLZ group. The OLZ group maintained significantly greater MAP than all the treatment groups (I, III, IV, V, and VI) from 45 to 90 days (p < 0.001), underscoring the persistent hypertensive effect of OLZ over time. MAP was significantly lower in groups V and VI than in the OLZ group after 15, 30 (p < 0.05), and 45–90 days (p < 0.001), highlighting the ability of probiotics to counteract OLZ-induced hypertension (supplementary data Table 2 and Fig. 3).

After 30 days of OLZ treatment, there was a significant increase in TC levels compared to those in the N (p < 0.05), PM (p < 0.05), PH (p < 0.05), and both TM and TH (p < 0.001) groups. After 45 days, the OLZ group continued to exhibit a significant increase in TC levels compared to those of the N (p < 0.05), TM (p < 0.05), PM, and PH (p < 0.001) groups. The pattern persisted at 60, 75, and 90 days, with the OLZ group showing significantly higher cholesterol levels than the normal control group and all other treatment groups (p < 0.001). The sustained cholesterol-lowering benefit of probiotic treatment, alone or in conjunction with OLZ (p < 0.001), was evident throughout the study (Table 1).

**Table 1:** Comparison of lipid profiles {TC, TG, and HDL-C levels} (mg/dl) of various experimental groups at baseline and at the end of 90 days

Group (n = 6)	TC—baseline	TC—90 days	TG—baseline	TG—90 days	HDL-C baseline	HDL-C 90 days
Group I: normal control (N)	124.07 ± 1.39	125.99 ± 1.56	86.81 ± 2.87	89.48 ± 2.26	64.55 ± 2.99	65.98 ± 2.84
Group II: OLZ (O)	124.16 ± 1.98	163.16 ± 4.07μδ	86.17 ± 2.93	127.57 ± 2.85μδ	62.26 ± 1.92	49.86 ± 2.14κλ#£
Group III: probiotic I (probiotic low dose)—PM	126.46 ± 1.79	108.90 ± 1.87γ	87.74 ± 2.68	87.41 ± 1.16Å	58.97 ± 2.55	75.53 ± 2.02γ
Group IV: probiotic II (probiotic high dose)—PH	123.76 ± 1.82	112.27 ± 1.34γ	85.17 ± 2.78	82.07 ± 1.59Å	60.99 ± 2.97	70.50 ± 2.82γ
Group V: OLZ + probiotic-I (TM)	120.43 ± 1.84	118.32 ± 2.08μκ@	86.56 ± 1.73	90.10 ± 2.37Å	58.95 ± 2.90	62.50 ± 2.88*κλ
Group VI: OLZ + probiotic-II (TH)	120.67 ± 1.19	123.08 ± 1.52μκλ	85.60 ± 2.48	89.30 ± 1.70Å	60.86 ± 2.38	58.55 ± 1.41κλ

Values are represented as the mean ± SEM, n: number of rats in each group; SEM: standard error of mean, TC values are expressed in mg/dl

*Compared to (vs) OLZ (p < 0.05); Å: vs O (p < 0.001); μ: vs N (p < 0.001); κ: vs probiotic-I (p < 0.001); λ: vs probiotic-II (p < 0.001); δ: vs probiotic (I and II), TM and TH (p < 0.001), #: vs N (p < 0.05); £: vs TM (p < 0.05); γ: vs N, O, TM, TH (p < 0.001)

After 15 days of OLZ treatment, there was a significant increase in TG levels compared to those in the PM and TM groups (p < 0.05). After 30, 45, 60, 75, and 90 days of treatment, OLZ significantly increased the TG levels compared to those in all other groups (p < 0.001). Both probiotic-only treated groups consistently exhibited significant reductions in TG levels, indicating a sustained antihyperlipidemic effect. The TM and TH treatments effectively reduced TG levels, suggesting that probiotics are more effective at mitigating the hyperlipidemic effects of OLZ over an extended period.

After 30 days, OLZ treatment significantly decreased HDL-C levels compared to those in both the low- and high-dose probiotic groups (p < 0.05). Similarly, compared with those in the OLZ group, the HDL-C levels in the TM and TH groups were significantly greater (p < 0.001). After 45 days of treatment, the data showed that both the low- and high-dose probiotic groups experienced a significant increase in HDL-C levels compared to groups I, II, V, and VI, with p < 0.001. This trend of significant improvement continued for both probiotic-only treated groups at the end of 60, 75, and 90 days, consistently showing higher HDL-C levels compared to all other groups, with significance noted at p < 0.001 across all these time points.

Conversely, compared with OLZ alone, the combination of OLZ with either low-dose or high-dose probiotics increased HDL-C levels at 45 days (p < 0.001). This pattern of increase persisted at 60, 75, and 90 days, indicating a beneficial effect of probiotics when used in conjunction with OLZ, suggesting sustained improvements in HDL-C levels with probiotic cotreatment.

**Fig. 4:**
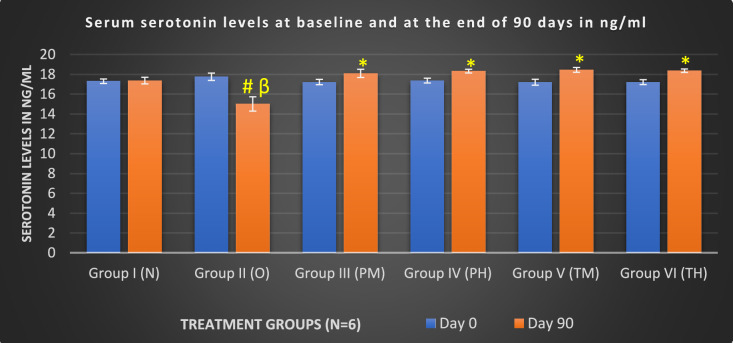
Comparison of serum serotonin levels at baseline and at the end of 90 days of treatment in various treatment groups. *Compared to (vs) O (p < 0.05); β: vs groups (PM, PH, TM and TH) (p < 0.05); #: vs N (p < 0.05)

**Fig. 5:**
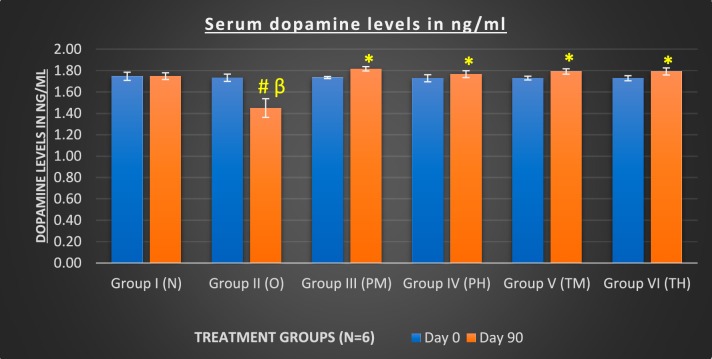
Comparison of serum dopamine levels at baseline and at the end of 90 days of treatment in various treatment groups. *Compared to (vs) O (p < 0.05); β: vs PM, PH, TM and TH groups (p < 0.05); #: vs N (p < 0.05)

After the 90-day study, OLZ treatment significantly decreased the serum serotonin and serum dopamine levels in rats (p < 0.05) compared to those in the probiotics group (both low- and high-dose) when given alone and in combination with OLZ. Conversely, treatment with both low and high doses of probiotics alone or in combination with OLZ significantly increased serum serotonin and serum dopamine levels (p < 0.005) compared to those in the OLZ-only group, indicating the effectiveness of long-term use of probiotics in managing the OLZ-induced changes in serotonin and dopamine levels that impact MS (Figs. 4 and 5).

### Histopathological evaluation of the intestine

**Fig. 6:**
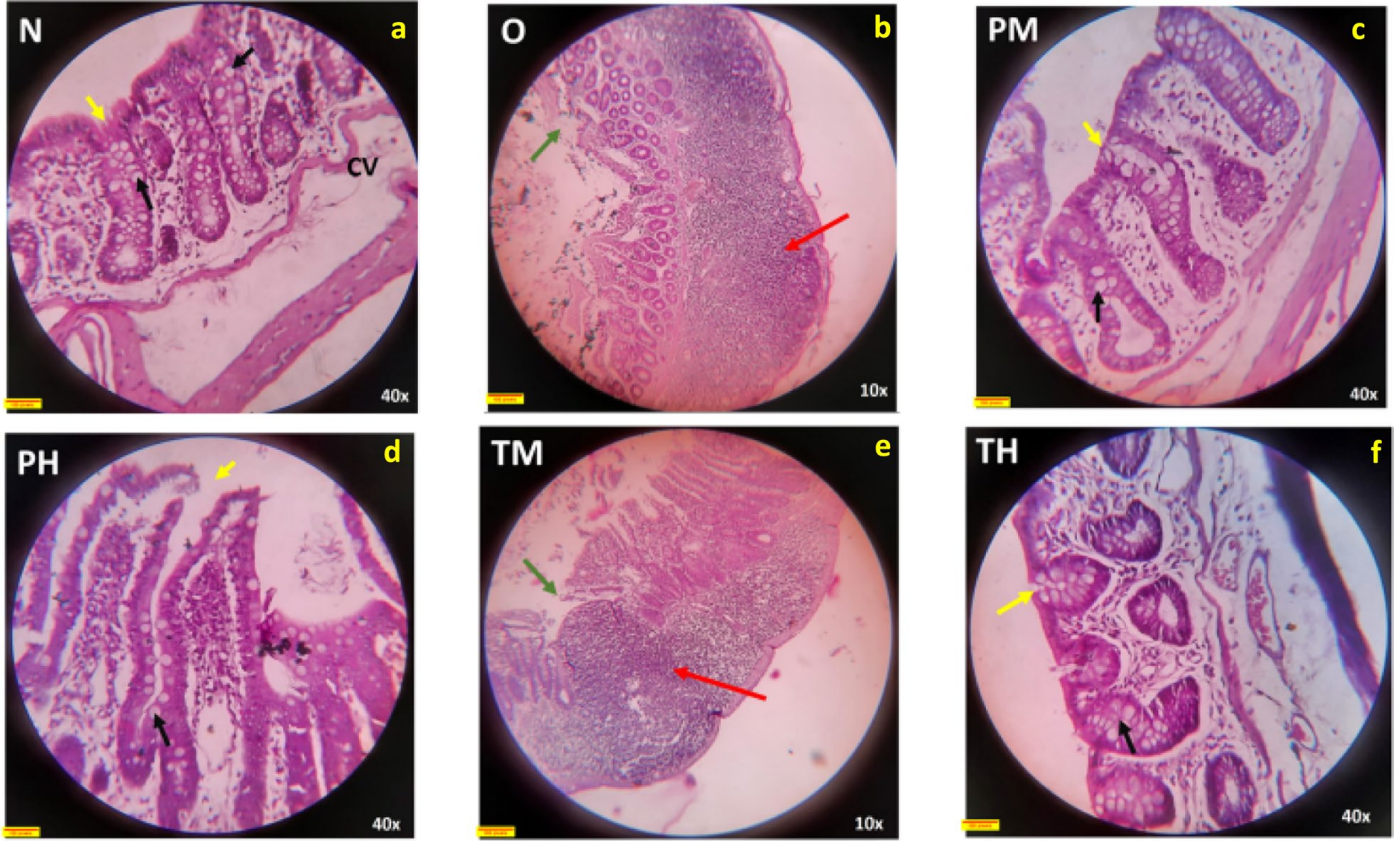
H&E-stained rat intestine under ×40 magnification showing changes (arrows) in the normal control (N) (**a**), probiotic low dose (PM) (**c**), probiotic high dose (PH) (**d**), OLZ + probiotic low dose (TM) (**e**), OLZ (O) (**b**), and OLZ + probiotic high dose (TH) (**f**) groups at the end of 90 days

H&E-stained cross-sections of the colon were observed under a light microscope at 40× magnification. The structure of the mucosal and submucosal regions was considered for qualitative assessment. The N, PM, PH, and TH groups exhibited normal colon epithelium with intact surface epithelium and intestinal crypts (shown with yellow arrow) with abundant goblet cells (black arrow). The submucosa looked normal, with loose connective tissue and blood vessels (Fig. 6).

The colonic mucosa looked highly abnormal in the O (Fig. 6b) and TM (Fig. 6e) groups. There were several indications of superficial mucosal necrosis. Intestinal crypts appeared damaged and reduced in number in some places (white arrow). At many places, massive inflammatory cell infiltration into the lamina propria, submucosa, and surrounding smooth muscle fibres of the muscularis mucosa was observed (red arrows) (Fig. 6b, e). Other histopathological features of ischaemic colitis, such as intestinal crypt injury, crypt dropout, lamina propria hyalinization, and vascular congestion, were also observed in some regions.

**Fig. 7:**
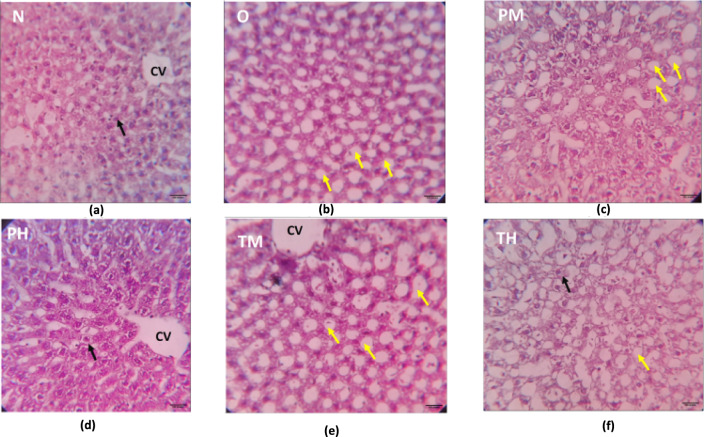
H&E-stained rat hepatic tissue under ×40 magnification showing changes in the normal control (N) (**a**), OLZ (O) (**b**), probiotic low-dose (PM) (**c**), probiotic high-dose (PH) (**d**), OLZ + probiotic low-dose (TM) (**e**), and OLZ + probiotic high-dose (TH) (**f** groups at the end of 90 days of treatment; CV is the central vein of the liver, the black arrow indicates normal hepatocytes, and the yellow arrow indicates fatty liver cells

Photomicrographs of H&E-stained liver tissue as observed under a light microscope (40× magnification). Two major components of liver structure, the hepatocellular architecture and biliary system, were considered for qualitative assessment. Notably, the hepatic architecture of the N, PM, and PH groups was normal (the black arrow indicates normal hepatocytes). Group O, TM, and TH showed features of fatty liver (the yellow arrows indicates fatty liver cells) (Fig. 7).

#### Results of qualitative assessment of H&E-stained liver tissue

The normal control group (Fig. 7a) exhibited a normal liver structure with hexagonal hepatic lobules, a central vein, and a typical arrangement of hepatic cords radiating from the central vein. Portal triads were also normal in their location and pattern. Group O (Fig. 7b) exhibited abnormal hepatic architecture. Large areas of steatosis (fatty liver) and inflammatory cell collection indicate pathology leading to MS. The PM and PH groups (Fig. 7c, d) mostly exhibited a normal hepatocyte structure and architecture without fatty liver or inflammatory cells. The TM group (Fig. 7e) showed predominantly normal hepatic architecture; some areas of tissue demonstrated features of fatty liver. The TH group (Fig. 7f) mostly exhibited normal hepatocyte structure and architecture. However, some areas showed features of mild fatty cells in the liver.

#### 16S rRNA metagenomic analysis—modifications in the gut microbiome composition

**Fig. 8:**
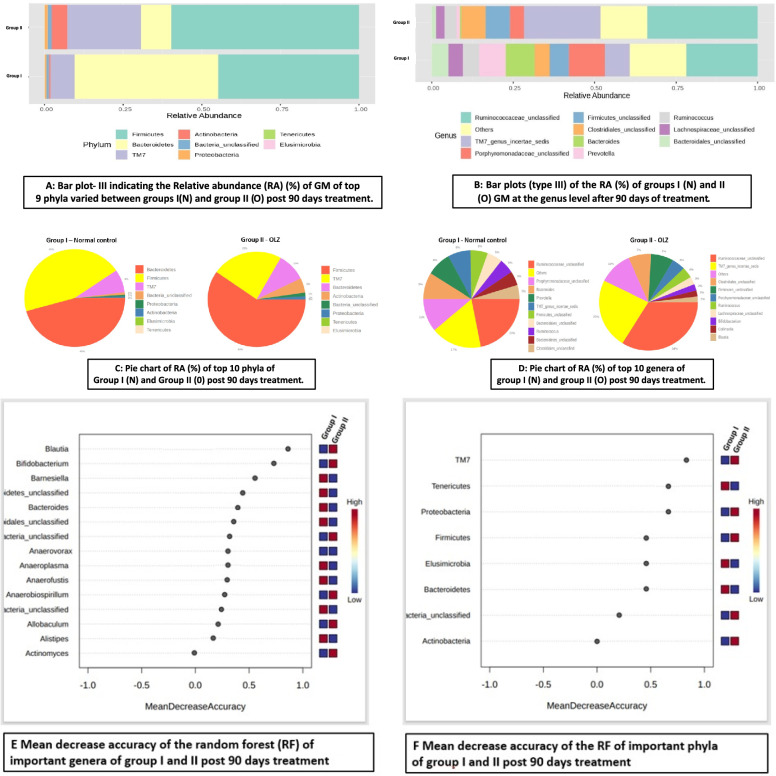
Bar plot-III (**A**, **B**) indicating that the relative abundance (RA) (%) of the GM of the top nine phyla (**A**) and ten genera (**B**) varied between group I (N) and group II (O) after 90 days of treatment; **C**, **D** are pie charts of the RA (%) of the top 8 phyla (**C**) and the top 10 genera (**D**) of group I and group II after 90 days of treatment (**E**)MDA plot of genera (group I vs. group II), (**F**) MDA plot of phyla (group I vs. group II

Taxonomic analysis was performed using a bar plot (type III) of the relative abundance (RA) (%) of phyla and genera in the GM between various groups after 16S rRNA metagenomic analysis of the rat fecal samples. RA bar plots of the phylum comparisons of the treatment groups are given below:

As shown in Fig. 8A, the taxonomic analysis bar plot (type III) indicated that the RA (%) of the GM of the top nine phyla varied between group I and group II after 16S rRNA metagenomic analysis of the rat fecal samples. Among these phyla, the *Firmicutes* group (60%) was predominant in group II, followed by the *Saccharibacteria* (*TM7*) (23%), *Bacteroidetes* (10%), *Actinobacteria* (5%), other bacteria (1%), and *Proteobacteria* (1%) groups. In group I, the predominant phyla were the *Bacteroidetes* (46%), *Firmicutes* (45%), *TM7* (8%), other bacteria (1%), and *Proteobacteria* (1%) (Fig. 8C).

Taxonomic analysis indicated that the RA (%) of the 43 genera in the GM varied between groups I and II. Figure 8B–D shows the 10 most abundant genera and the genera of the other organisms in both groups. Among these genera, *Ruminococcaceae* (34%) was the predominant genus in group II, followed by *TM7*_genus_*incertae*_*sedis* (23%), others (11%), *Clostridiales* and *Firmicutes* (7% each), *Porphyromonadaceae* and *Ruminococcus* (4% each), *Lachnospiraceae* (3%), *Bifidobacterium* (2%), *Collinsella* (2%), and *Blautia* (2%). In the normal control group, *Ruminococcaceae* (22%) was the most prevalent genus, followed by other bacteria (17%), *Porphyromonadaceae* (11%), *Bacteroides* (9%), *Prevotella* and *TM7*_genus_*incertae*_*sedis* (8% each), *Firmicutes* (6%), *Bacteriodales*, *Ruminococcus*, *Bacteroidetes* and *Clostridiales* (5% each).

The MDA plot of genera (group I vs. group II) (Fig. 8E) illustrates that *Blautia, Bifidobacterium*, *Barnesialla*, *Bacteroidetes*, *Bacteroides*, *Bacteroidales*, *Anaerovorax, Anaeroplasma*, *Anaerofustis*, *Anaerobiospirillum*, *Allobaculum*, *Alistipes*, and *Actinomyces* are important genera for differentiating/classifying these two groups. Lower *Blautia*, *Bifidobacterium*, *Anaerovorax*, *Anaerobiospirillum*, *Allobaculum*, and *Actinomyces abundances are characteristic of group I*, whereas higher *Barnesiales*, *Bacteroidetes*, *Bacteroides*, *Bacteroidales*, *Anaeroplasma*, *Anaerofustis*, and *Alistipes* abundances are characteristic of group II. Both groups of organisms can be identified using the levels of these genera.

The MDA plot of phyla (group I vs. group II) (Fig. 8F) illustrates that *TM7*, *Tenericutes*, *Proteobacteria*, *Firmicutes*, *Elusimicrobia*, *Bacteroidetes*, and *Actinobacteria* are important phyla for differentiating/classifying these two groups. Lower *TM7*, *Proteobacteria*, *Firmicutes*, and *Actinobacteria* abundances and higher *Tenericutes*, *Elusimicrobia*, and *Bacteroidetes abundances* are characteristic of group I. The exact opposite is true for group II. Using the levels of these phyla, both groups of organisms can be identified. A higher MDA value indicates the importance of that taxa in predicting or differentiating the groups. The genera/phyla obtained in the MDA plots are essential for classifying the respective groups.

The genus comparison bar plots, MDA plots, and pie charts of the remaining group comparisons are provided in the supplementary files.

**Fig. 9:**
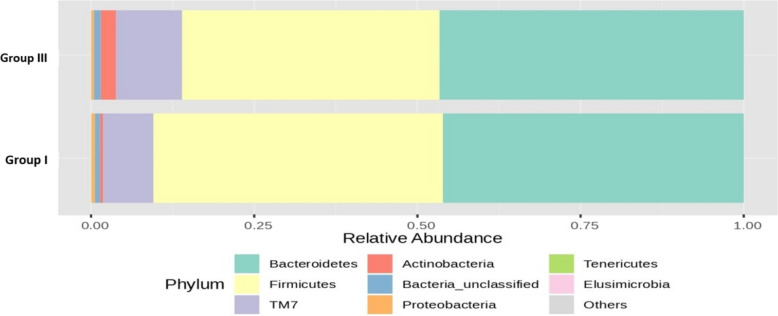
Bar plots of the RAs (%) of the GM phyla of group I (N) and group III (PM) after 90 days of treatment

The RA (%) of the GM of the top nine phyla varied between groups I and III after 16S rRNA metagenomic analysis of the rat faecal samples (Fig. 9). Among these phyla, *Bacteroidetes* (46%) was predominant in group I, followed by the *Firmicutes* group (44%), *TM7* (8%), other bacteria (1%), and *Proteobacteria* (1%). In group III, the predominant phyla were the *Bacteroidetes* (47%), *Firmicutes* (39%), *TM7* (10%), *Actinobacteria* (2%)*,* and other bacteria (1%).

**Fig. 10:**
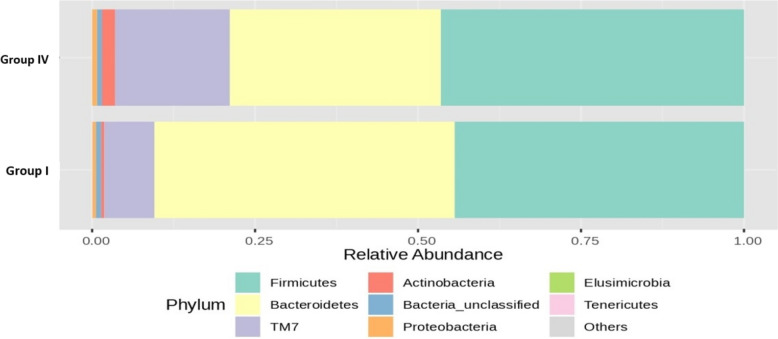
Bar plots of the RA (%) of the GM of groups I (N) and IV (PH) phyla after 90 days of treatment

The RAs (%) of the GM of the top nine phyla varied between groups I and IV after 16S rRNA metagenomic analysis of the rat faecal samples, as depicted in Fig. 10. Among these phyla, *Bacteroidetes* (46%) was the most abundant in group I, followed by *Firmicutes* (44%), *TM7* (8%), other bacteria (1%), and *Proteobacteria* (1%). In group IV, the most prevalent phylum was *Firmicutes* (47%), followed by *Bacteroidetes* (32%), *TM7* (18%), *Actinobacteria* (2%), other bacteria (1%), and *Proteobacteria* (1%).

**Fig. 11:**
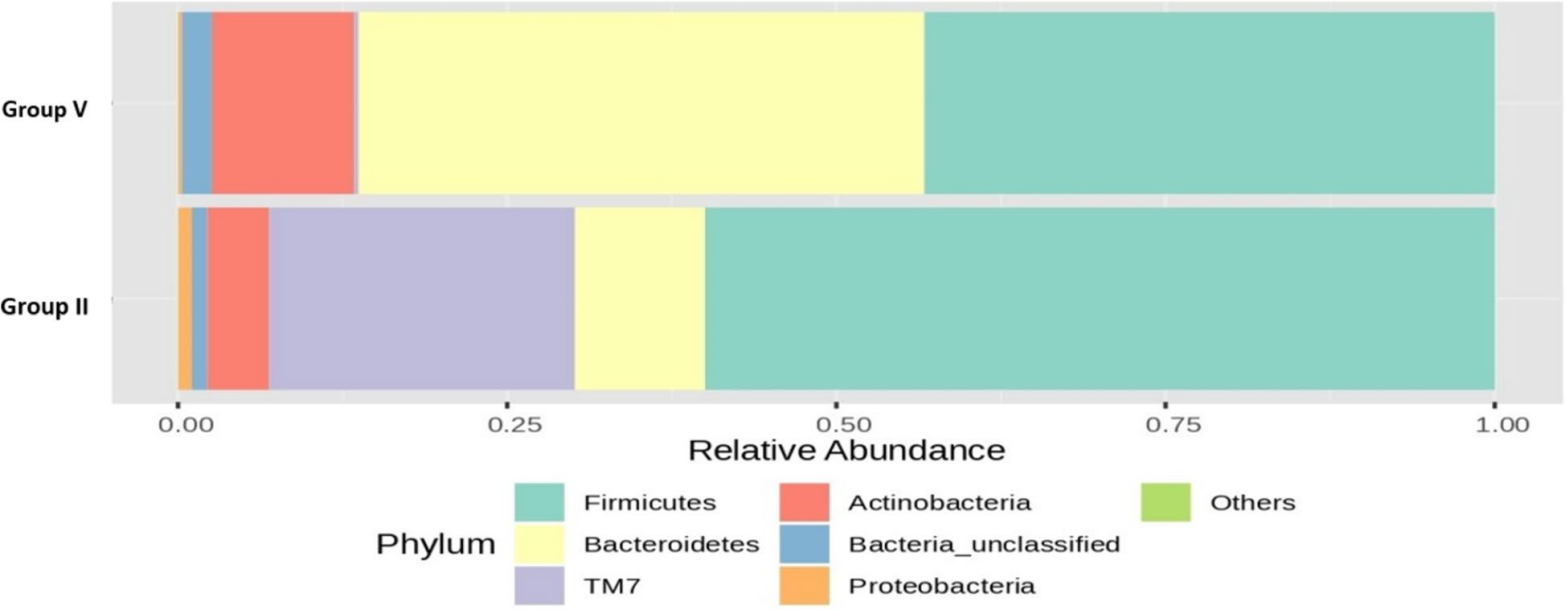
Bar plots of the RA (%) of the GM of the group II (O) and group V (TM) phyla after 90 days of treatment

Figure 11 depicts the bar plot of the RA (%) of the GM of phyla that varied between groups II and V after 16S rRNA metagenomic analysis of the rat faecal samples. After supplementation with a low dose of probiotics along with OLZ, the abundance of *Firmicutes* decreased (43% compared with 60% in group II), with a substantial 33% increase in the abundance of *Bacteroidetes* in group V (43% compared with group V vs. 10% compared with group II). There was also an increase in *the abundance of Actinobacteria* (11% compared with group V vs. 5%—group II) and a decrease in the abundance of Actinobacteria in *TM7* (< 1%—group V vs. 23%—group II) compared with those in the OLZ group.

**Fig. 12:**
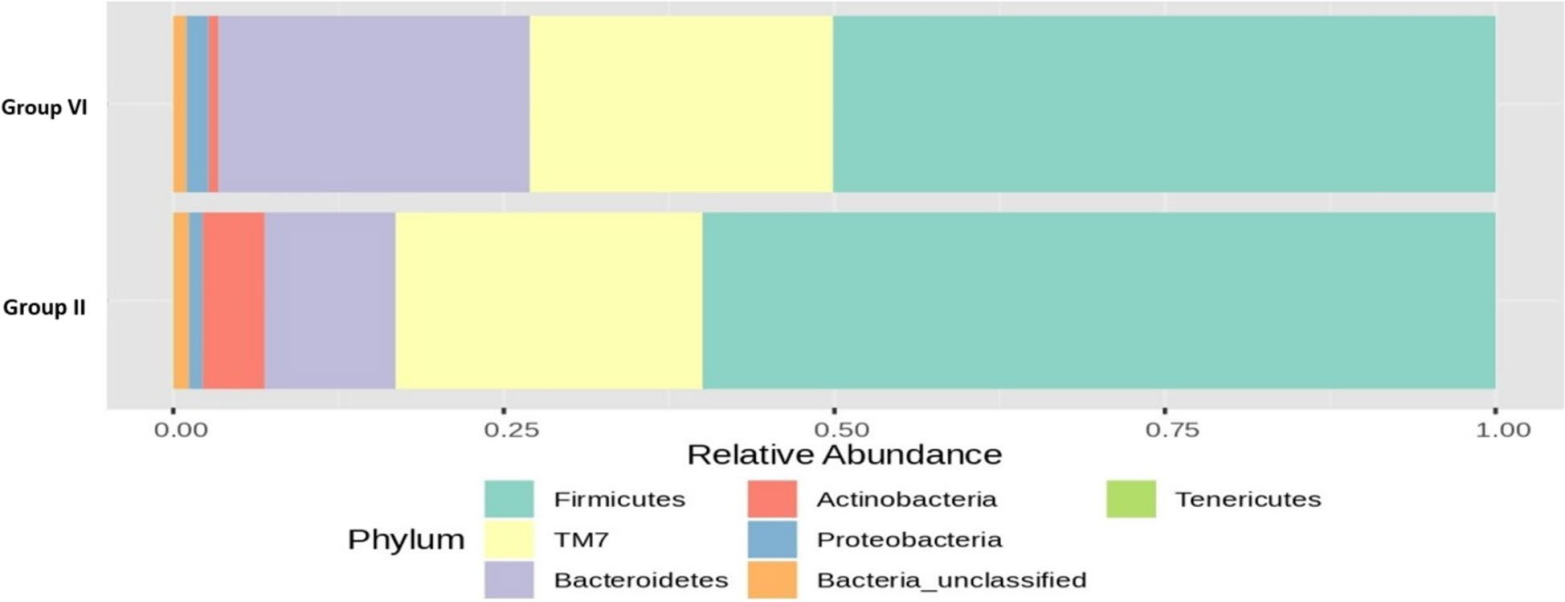
Bar plots of the RA (%) of the GM of the group II (O) and group VI (TH) phyla after 90 days of treatment

The RA (%) of the GM of the top seven phyla varied between groups II and VI after 16S rRNA metagenomic analysis of the rat faecal samples, as depicted in Fig. 12. There was a noteworthy 14% increase in *Bacteroidetes* and a minor 1% increase in the *Proteobacteria* phylum in group VI, whereas 10% *Firmicutes* and 4% *Actinobacteria* were reduced compared to those in group II.

**Fig. 13:**
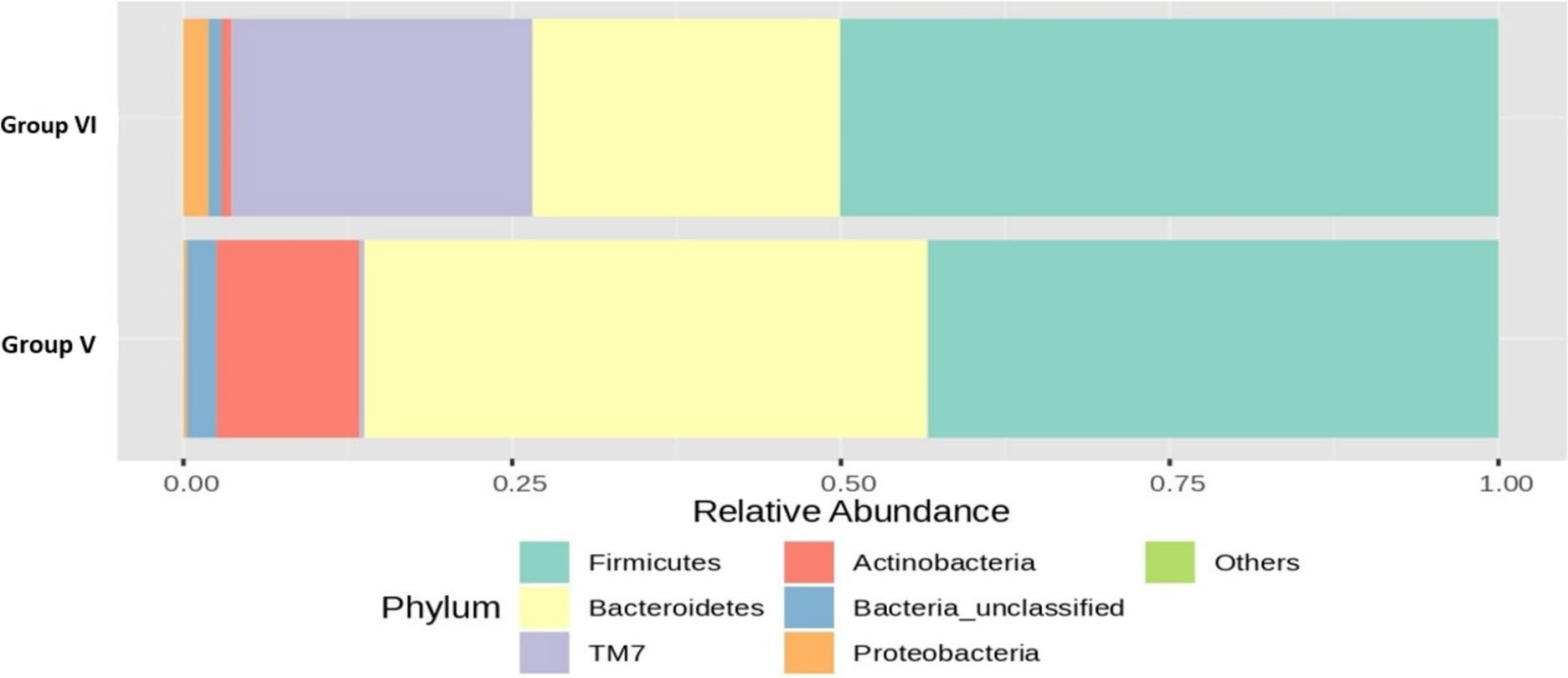
Bar plots of the RA (%) of the GM of group V (TM) and group VI (TH) at the phylum level after 90 days of treatment

Figure 13 shows that the RA (%) of the GM of the top seven phyla varied between groups V and VI after 16S rRNA metagenomic analysis of the rat faecal samples. There was a 23% and 7% increase in the RA of *TM7* and *Firmicutes, respectively,* in group VI compared to group V. *Bacteroidetes* and *Actinobacteria* were increased by 19% and 10%, respectively, in the low-dose group compared to the high-dose group, highlighting the dose-dependent advantage of probiotics in inducing the phyla that are beneficial for preventing the symptoms of MS.

**Fig. 14:**
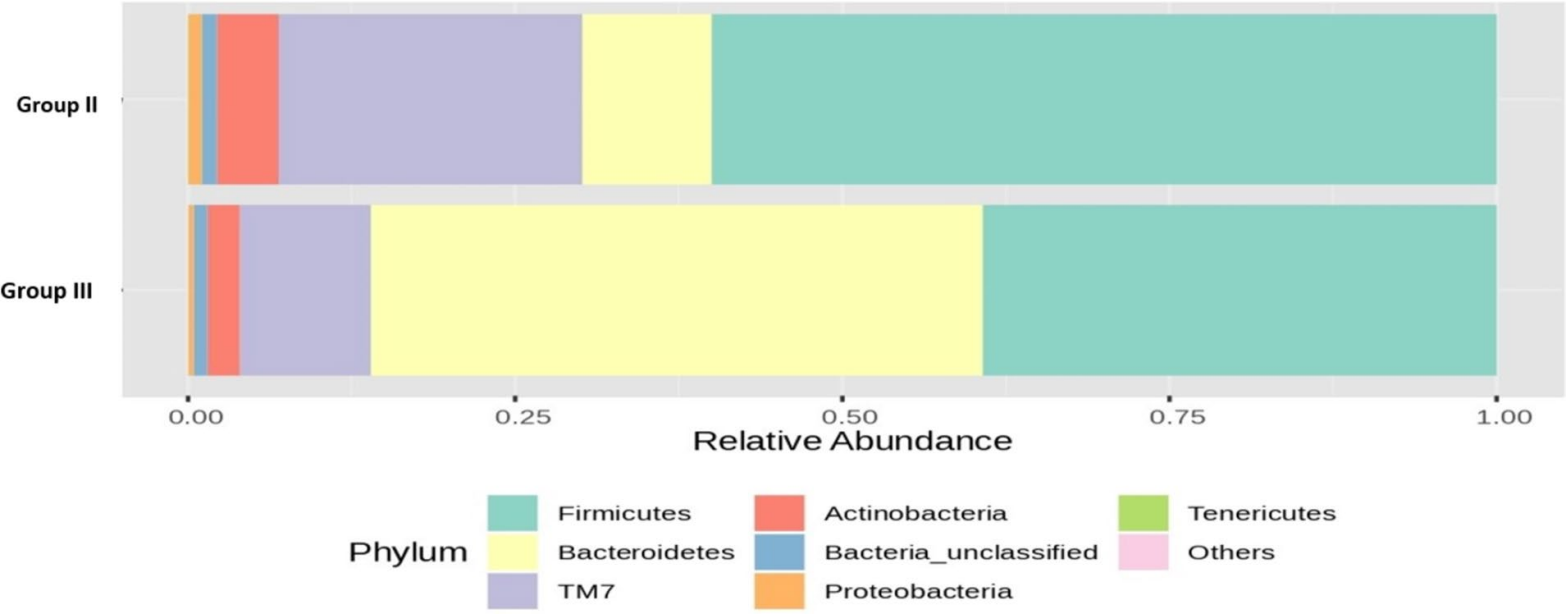
Bar plots of the RA (%) of the GM of group II (O) and group III (PM) at the phylum level after 90 days of treatment

Figure 14 shows the RA (%) of the GM across the top phyla, highlighting differences between groups II and III following 16S rRNA metagenomic analysis of the rat fecal samples. Notably, there was a 37% increase in the RA of the beneficial *Bacteroidetes* phylum in the group administered a low dose of probiotics compared to that in the OLZ group. This finding underscores the potential of probiotics for promoting MGBA homeostasis. Conversely, the OLZ group exhibited 21% and 13% increases in the *Firmicutes* and *TM7* phyla, respectively. These changes highlight the association of OLZ with dysbiosis and the disruption of intestinal integrity, potentially leading to MS.

**Fig. 15:**
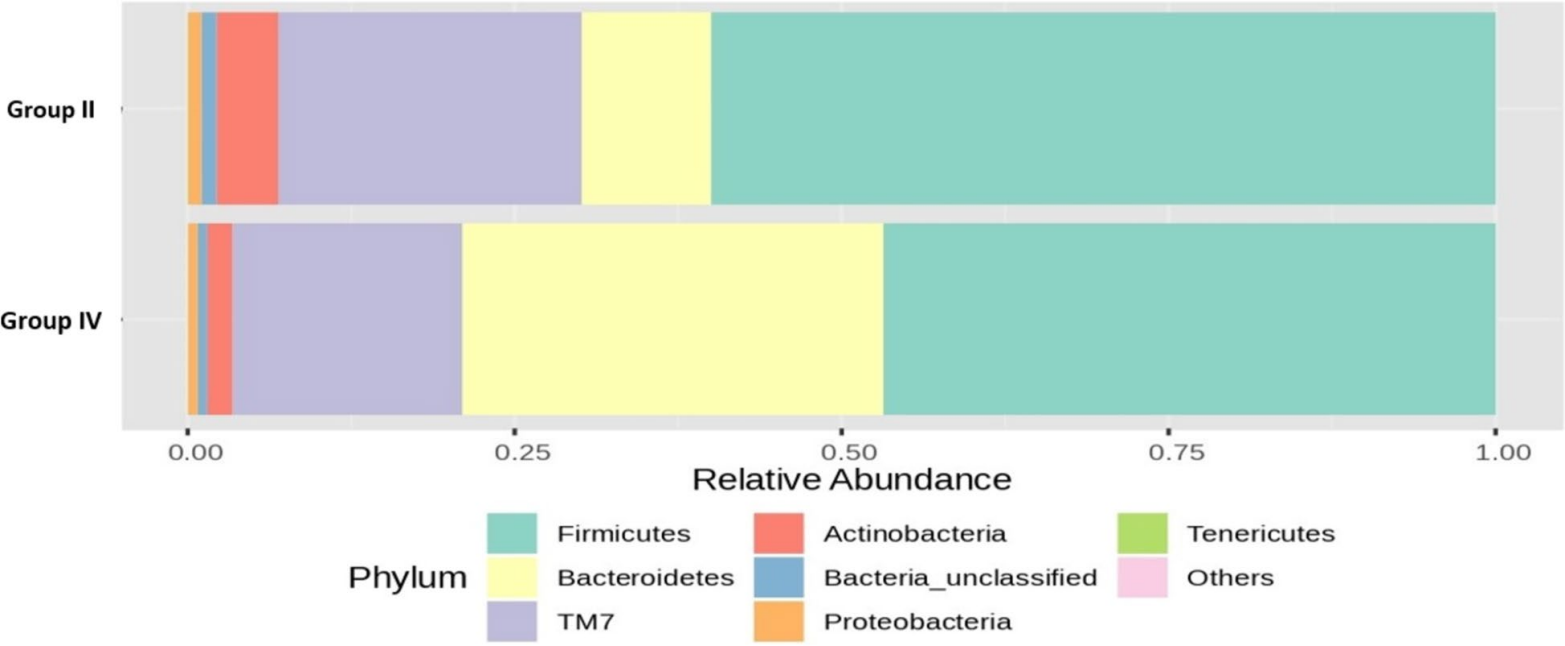
Bar plots of the RA (%) of the GM of groups II (O) and IV (PH) at the phylum level after 90 days of treatment

The bar plot (Fig. 15) shows the RA (%) across the top phyla, highlighting differences between the GM of groups II and IV following 16S rRNA metagenomic analysis of rat fecal samples. Notably, there was a 22% increase in the RA of the beneficial *Bacteroidetes* phylum in the group administered a high dose of probiotics compared to that in the OLZ group. This finding underscores the potential of probiotics in promoting MGBA equilibrium. In contrast, the OLZ group exhibited 13%, 6%, and 3% increases in the *Firmicutes*, *TM7*, and *Actinobacteria* phyla, respectively. These changes highlight the association of OLZ with dysbiosis and the loss of gut barrier integrity, potentially leading to MS.

**Fig. 16:**
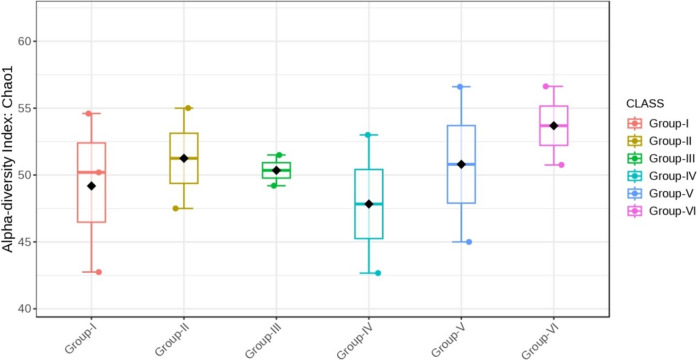
Comparison of alpha diversity among microbiome of different genera using Chao1 (p < 0.05, Kruskal–Wallis test)

**Fig. 17:**
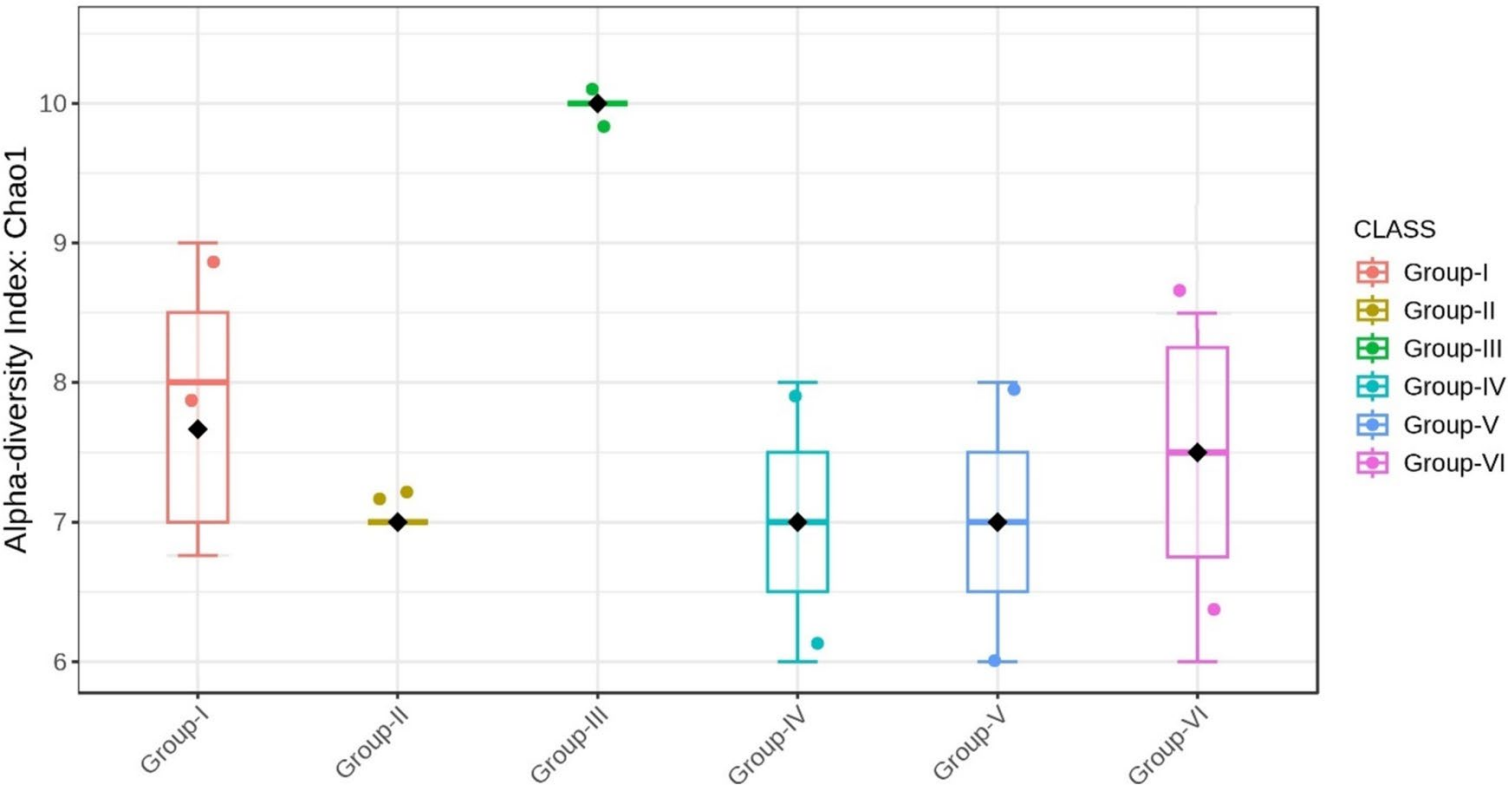
Comparison of alpha diversity among microbiome of different phylum using Chao1 (p < 0.05, Kruskal–Wallis test)

Alpha diversity was employed to assess both the richness and diversity of bacteria within a specific group. The species richness measured with the Chao1 index exhibited a significant difference between the groups (p < 0.05) (Figs. 16 and 17).

**Fig. 18:**
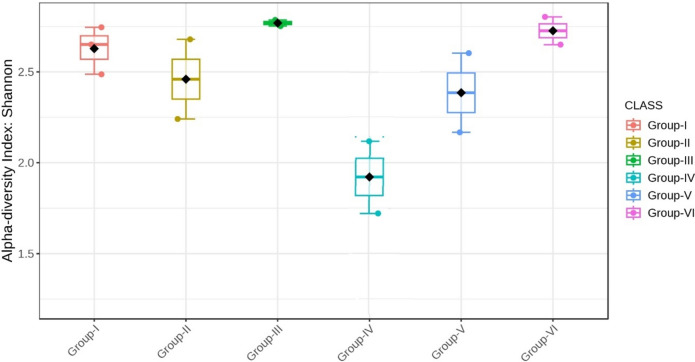
Comparison of alpha diversity among microbiome of different genera using Shannon index (p < 0.05, Kruskal–Wallis test)

**Fig. 19:**
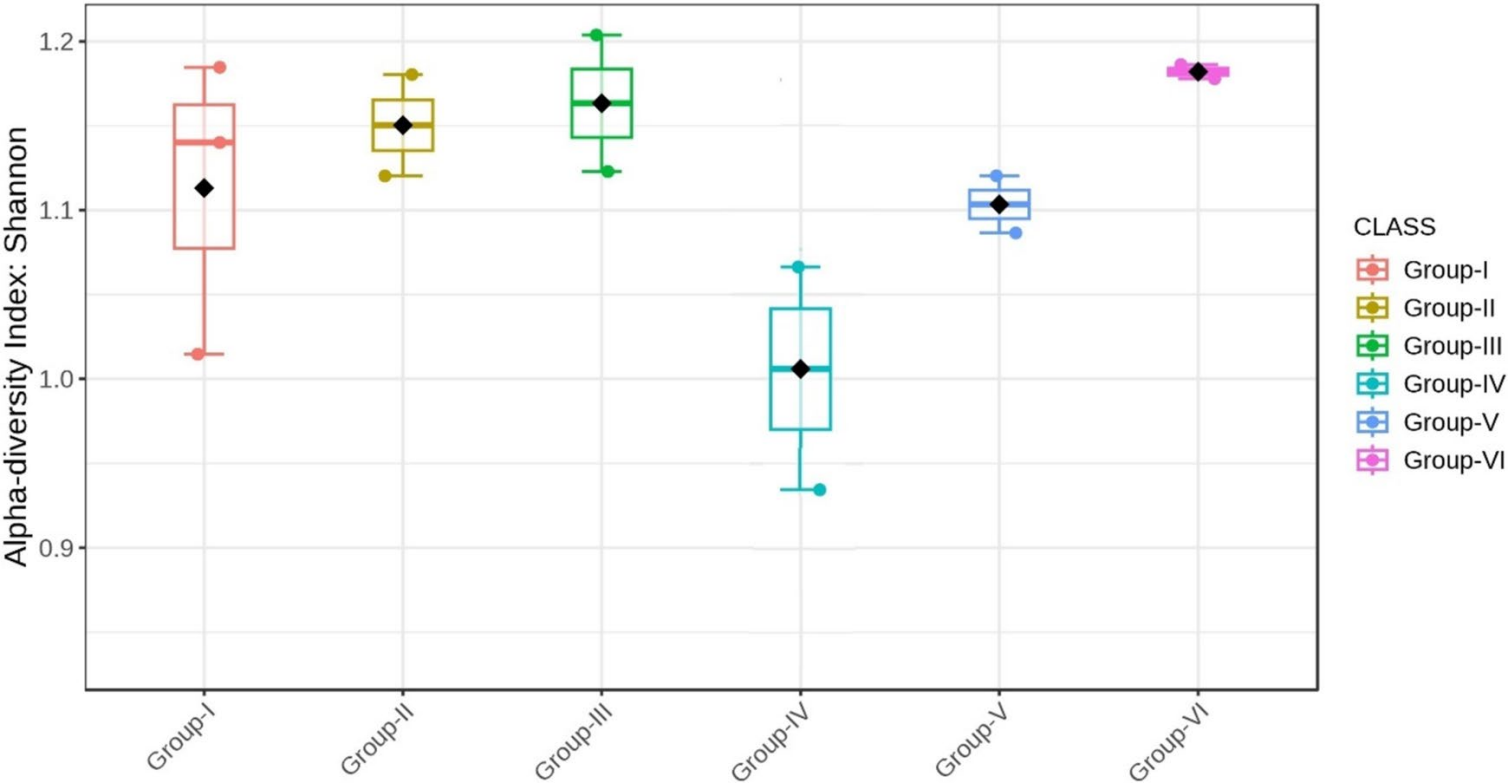
Comparison of alpha diversity among microbiome of different phylum using Shannon index (p < 0.05, Kruskal–Wallis test)

**Fig. 20:**
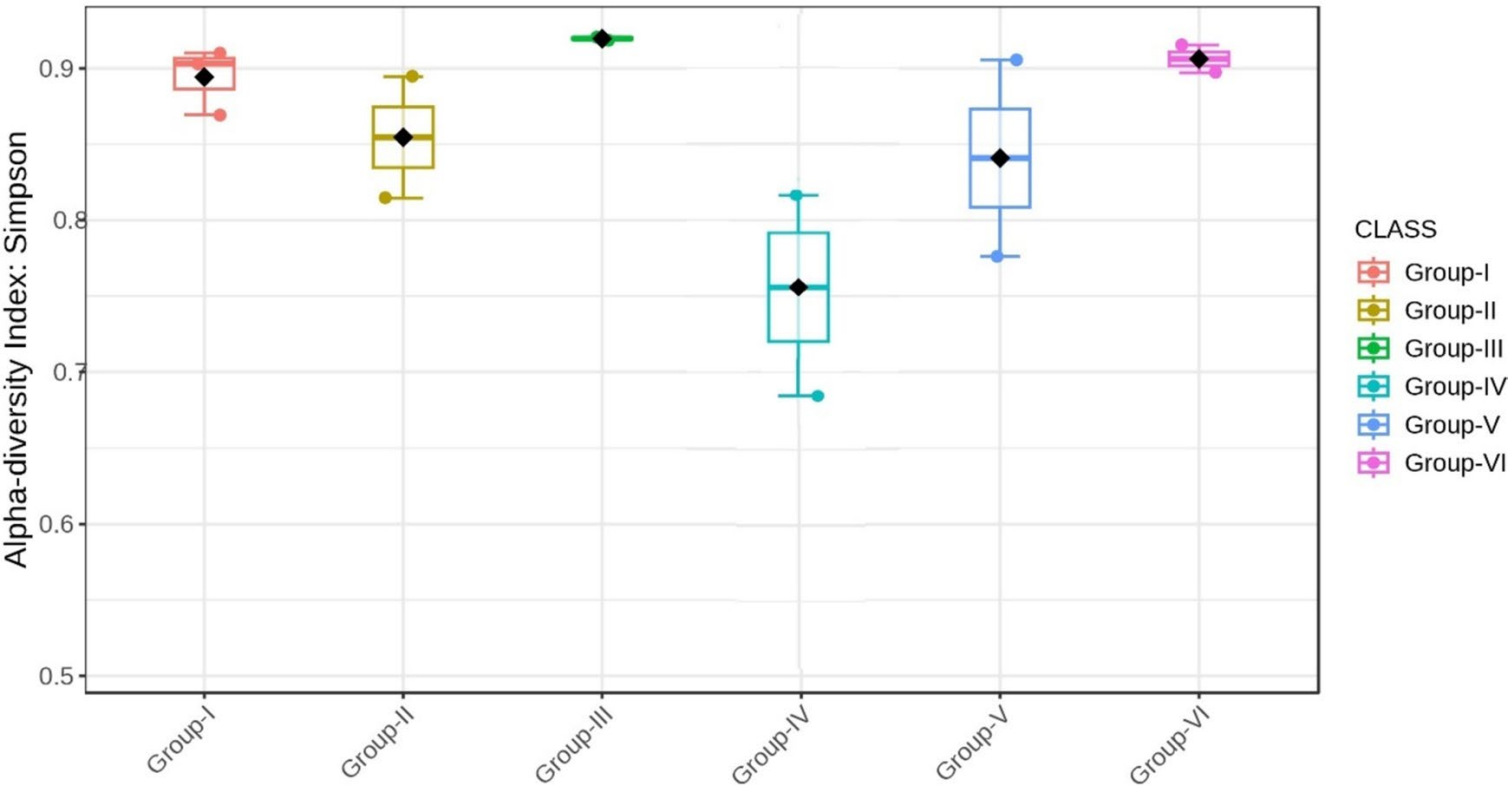
Comparison of alpha diversity among microbiome of different genera using Simpson index (p < 0.05, Kruskal–Wallis test)

**Fig. 21:**
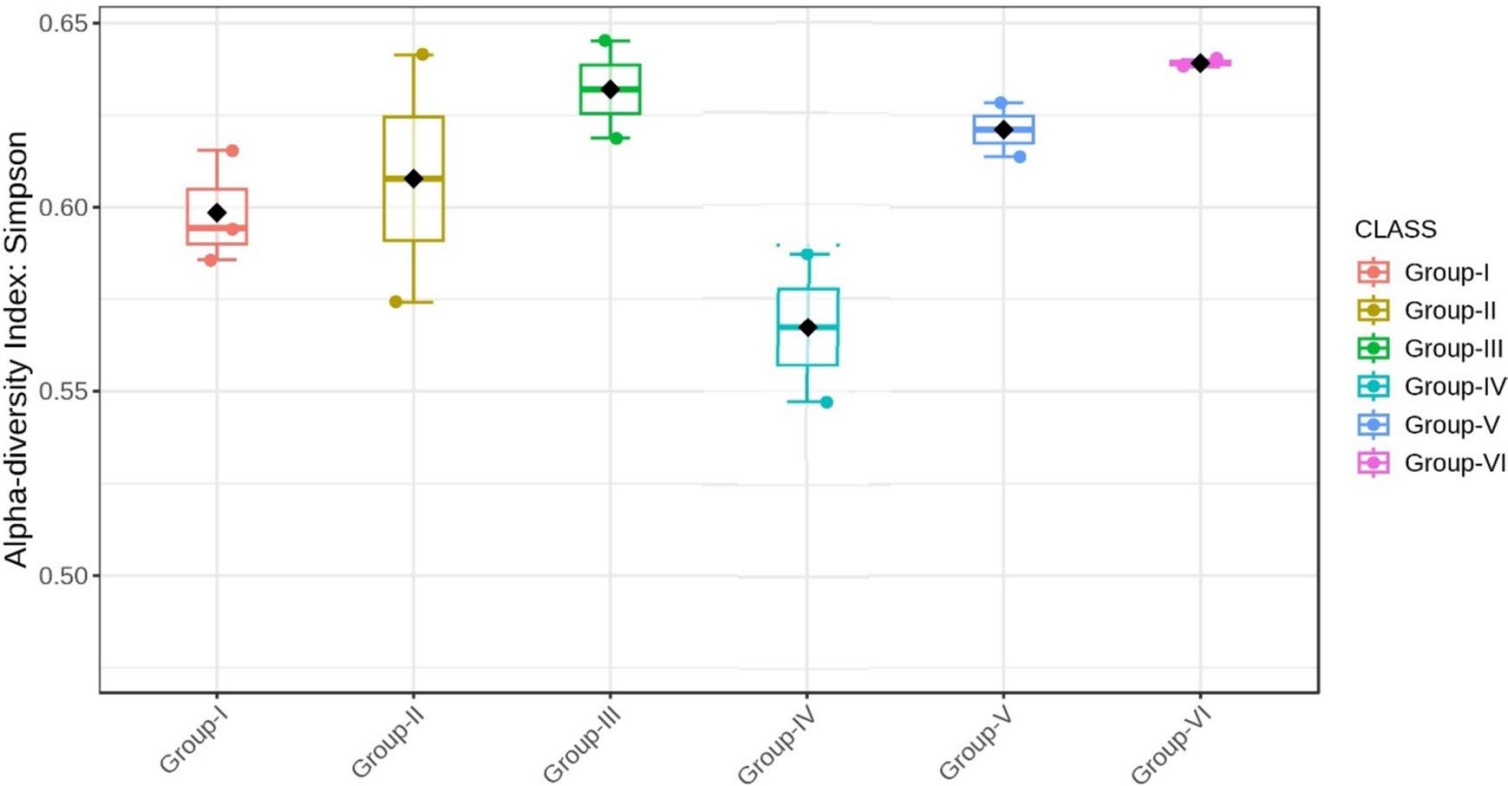
Comparison of alpha diversity among microbiome of different phylum using Simpson index (p < 0.05, Kruskal–Wallis test)

Almost all the groups demonstrated reduced alpha diversity, as evidenced by both Simpson (p < 0.001) and Shannon (p < 0.001) indices (Figs. 18, 19, 20, 21).

**Fig. 22:**
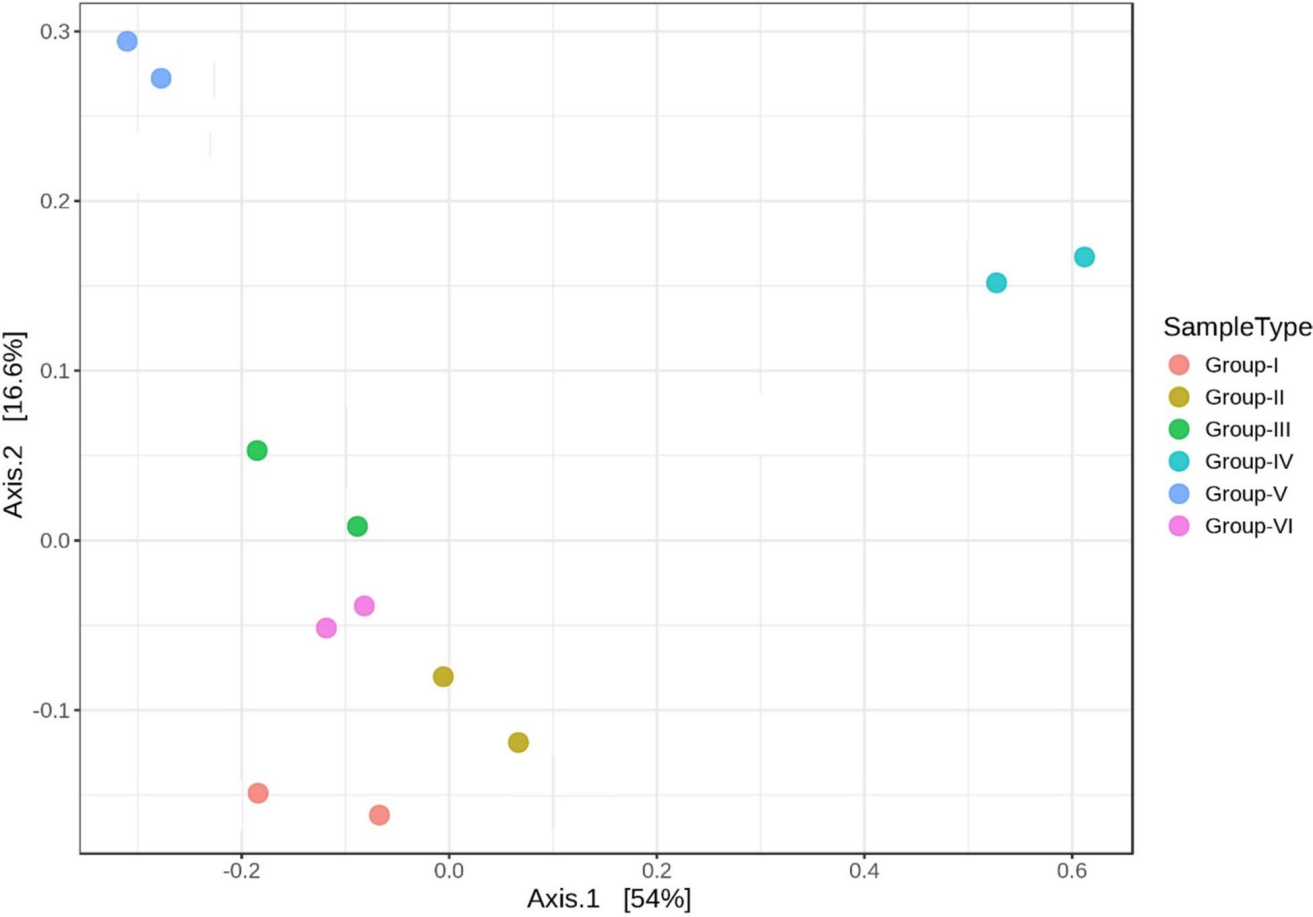
Principal coordinate analysis (PCoA) highlighting beta diversity of distinct bacterial communities of genera clustering among the groups (p < 0.01, PERMANOVA)

**Fig. 23:**
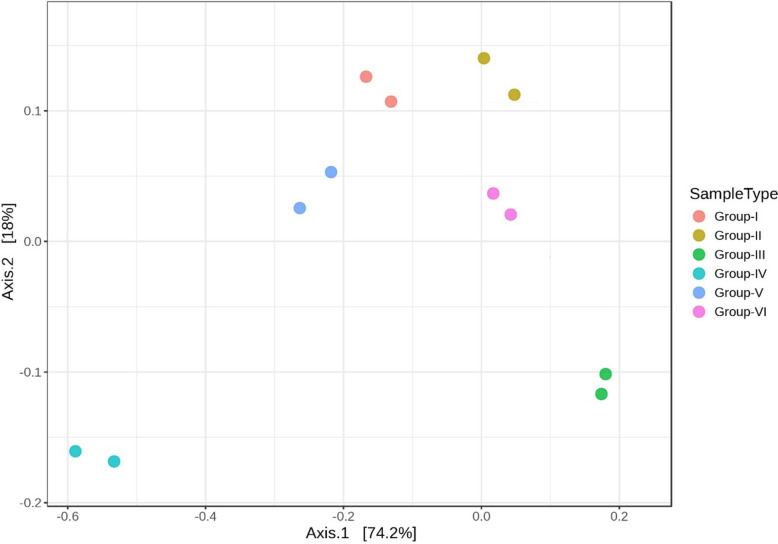
Principal coordinate analysis (PCoA) highlighting beta diversity of distinct bacterial communities of phylum clustering among the groups (p < 0.01, PERMANOVA)

**Fig. 24:**
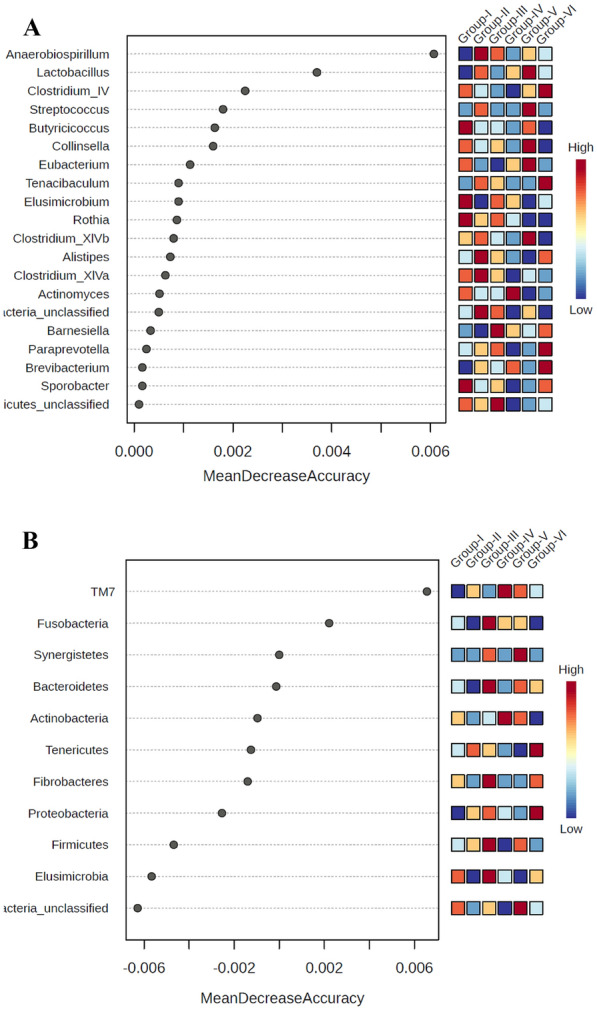
**A** The MDA plot of important genera as biomarkers among different groups. **B** The MDA plot of important phylum as biomarkers among different groups

The beta diversity analysis was carried out to understand how the microbial communities clustered among the six groups. Diversity profiles in samples were clustered and notably distinct from each group by the Principal Coordinate Analysis (Figs. 22 and 23). The compositional variations among the groups were found to be statistically significant (p < 0.01) based on the PERMANOVA test (Fig. 24).

## Discussion

This study aimed to evaluate the effect of probiotics on OLZ-induced MS in an animal model. In the present study, the administration of OLZ for 3 months led to weight gain, an increased lipid profile, increased blood pressure, and alterations in the GM, which are the etiological factors responsible for the induction of MS. This effect is attributed to the mechanism of action of OLZ, which involves the antagonism of central serotonin (5-HT_2C_) and H_1_ receptors, which are particularly implicated in its metabolic side effects, including increased appetite and weight gain and the antagonism of dopamine receptors, leading to increased appetite and reduced satiety [32–34]. These receptors play crucial roles in energy homeostasis and appetite regulation, and their blockade is associated with enhanced appetite and decreased energy expenditure.

Previous studies have demonstrated the metabolic side effects of OLZ, including weight gain, increased risk of hypertension [35], and dyslipidemia [36]. This study’s results align with these findings by highlighting the significant increase in weight, blood pressure, and lipids following OLZ treatment. The present study revealed that probiotic treatment mitigated OLZ-induced weight gain, hyperlipidemia, and hypertension.

The proposed hypothesis for OLZ-induced MS could be due to an alteration in the GM observed in the OLZ-treated group, which could lead to dysbiosis. Dysbiosis can damage the integrity of the gut barrier. A leaky gut might allow beneficial and harmful bacteria to migrate, further damaging the blood‒brain barrier and altering neurotransmitters such as serotonin and dopamine through modulation of MGBA, as shown in the OLZ-treated group. In contrast, treatment with probiotics led to an increase in beneficial microorganisms and a decrease in harmful bacteria/known to induce MS and psychosis. These findings highlight the vital role of probiotics in modulating the MGBA and alleviating OLZ-induced MS. The findings from our present 90-day study also showed that the decrease in serum serotonin and dopamine levels in rats treated with OLZ was reversed by treatment with probiotics.

Serotonin can influence carbohydrate and fat intake, which are critical factors in the development of obesity and MS. Enteroendocrine cells produce serotonin to activate vagal afferent fibres while expressing Toll-like receptors to detect microbes. By controlling gastrointestinal functions, they can indirectly affect vagal afferent fibres and regulate food intake [37]. Through the production of SCFAs, the GM can influence serotonin synthesis in the gut. The GM controls 64% of intestinal and 49% of serum 5-HT concentrations [38]. Dopamine dysregulation has been linked to obesity, with reduced dopamine receptor availability associated with increased body mass index (BMI) and obesity depression [39]. Dopamine, however, is essential for the reward system and motivation, including food intake. Dopamine dysregulation has been linked to obesity, with reduced dopamine receptor availability associated with increased BMI and obesity [40]. OLZ blocks dopamine receptors and may induce weight gain partly through this mechanism [41]. The present study’s results align closely with the established serotonin and dopamine parameters, highlighting the mechanism through which probiotics and OLZ modulate serotonin and dopamine receptors. This alignment emphasises the critical role that these neurotransmitter systems play in mediating the effects of both probiotics and OLZ on body weight and MS. By interacting with these neurotransmitter systems, probiotics and OLZ influence appetite and energy homeostasis, potentially mitigating or exacerbating metabolic side effects. This insight into the interplay between probiotics, OLZ, and neurotransmitter systems opens new avenues for therapeutic interventions to manage antipsychotic-induced weight gain and metabolic disturbances.

In the CNS, dopamine can influence blood pressure through its action in the hypothalamus and brainstem regions, which are crucial for cardiovascular control. Dopamine acting on D_2_ receptors in these areas can inhibit sympathetic nerve activity, decreasing blood pressure [42]. In the kidneys, dopamine produced locally acts as a paracrine and autocrine factor to promote natriuresis and diuresis, primarily through D_1_-like receptors. This action facilitates sodium and water excretion, reducing blood volume and blood pressure [43]. Dopamine can also exert direct effects on vascular smooth muscle cells, and the outcome depends on the dopamine receptor subtype. Activation of D_1_-like receptors generally leads to vasodilation, reducing peripheral resistance and lowering blood pressure. Conversely, stimulation of D_2_-like receptors might have variable effects on blood vessel tone, potentially causing vasoconstriction in specific contexts [43, 44].

The present study compared the GM composition in normal control and OLZ-treated groups of rats. Taxonomic analysis revealed that the OLZ group had a greater abundance of *Firmicutes* and a notable presence of phyla such as *TM7* and *Bacteroidetes*, among others, compared to the control group, which predominantly included *Bacteroidetes* and *Firmicutes*, with lower proportions of *TM7*. The genera analysis further distinguished the two groups, with *Ruminococcaceae* being predominant in OLZ-treated rats, along with a significant presence of *TM7*_genus_*incertae*_*sedis* and others, in contrast to the microbial composition of the normal control group.

Linking these findings to MS, the alteration in GM composition, specifically the increase in the *Firmicutes*/*Bacteroidetes* ratio observed in the OLZ group, has been previously associated with obesity and MS. Enriching *Firmicutes* and the corresponding decrease in *Bacteroidetes* could promote energy harvest from the diet, contributing to obesity, a core component of MS [45]. Furthermore, specific genera such as *Ruminococcus* in the OLZ group have been connected to the production of short-chain fatty acids (SCFAs) through the fermentation of dietary fibres. SCFAs, particularly butyrate, are crucial for maintaining gut barrier integrity and modulating inflammation, and they are pivotal for the development of MS [46]. A significant increase in *Actinobacteria* compared to that in the N group can be correlated with the findings of a human study in which Actinobacteria was found to be increased in the faecal microbiota of patients with schizophrenia with MS but decreased in the faecal microbiota of patients with schizophrenia without MS, indicating its role in the pathophysiology of MS [47]. A plausible correlation between *Firmicutes* and tumor necrosis factor-α (TNF-α) was demonstrated by treating obese children with atypical antipsychotics that altered their GM; this increase in *Firmicutes* abundance was directly correlated with elevated TNF-α levels [48]. Similar to the OLZ-treated group in this study, people with obesity showed a decrease in the relative abundance (%) of bacteria belonging to the *Tenericutes* phylum and an increase in *Firmicutes* to reduce *Bacteroidetes* [49]. In conjunction with the present study, mice fed a Western diet heavy in sugar and cholesterol showed a decrease in the relative abundance of *Bacteroidetes* and *Actinobacteria* and an increase in the phyla *Firmicutes* and *Proteobacteria* [13]. These findings suggest that the intestinal microbiota composition is affected by many factors and that the outcome of these influences is subject to significant heterogeneity.

The combination of OLZ with low-dose probiotics resulted in a decrease in *Firmicutes* and an increase in *Bacteroidetes* and *Actinobacteria*. There was also a notable decrease in *TM7* and *Proteobacteria* compared to those in the OLZ group. Significant reductions were observed in the RA of *Ruminococcaceae*, *TM7*, and *Clostridiales* in the group receiving OLZ and probiotics. Conversely, there was an increase in *Prevotellaceae*, and the reduction in *Firmicutes* and the increase in *Bacteroidetes* may be beneficial. High *Firmicutes*/*Bacteroidetes* ratios have been associated with adverse health outcomes, including obesity and inflammation. Certain bacterial groups, such as *Firmicutes* and *Proteobacteria*, have been linked to the induction of MS due to their role in promoting inflammation, insulin resistance, and dyslipidemia, which were increased in the OLZ group, indicating the role of OLZ in inducing MS. An increased *Firmicutes* to *Bacteroidetes* ratio has been associated with obesity and other metabolic diseases [45].

Conversely, *Bacteroidetes* and specific genera such as *Prevotella* and *Blautia* are known to enhance gut barrier function, produce SCFAs such as butyrate, and modulate inflammation, thus potentially inhibiting MS development. Specifically, increases in *Bacteroidetes* have been correlated with improved metabolic profiles and reduced obesity [50]. *Actinobacteria*, particularly *Bifidobacterium* species, have been shown to exert beneficial effects on reducing obesity and insulin resistance through the modulation of gut permeability and inflammation [51].

The observed changes in microbial composition, specifically the increase in *Bacteroidetes* and *Actinobacteria*, along with a decrease in *Firmicutes*, suggest a shift towards a microbiota profile that could inhibit MS. *Bacteroidetes*, through the production of SCFAs, can improve gut barrier integrity and reduce inflammation [52]. On the other hand, the reduction in *Firmicutes* and specific proinflammatory genera, such as *TM7* and Proteobacteria, could diminish their contribution to inflammation and lipid metabolism dysregulation, factors central to MS pathophysiology.

The findings from the present study indicate that treatment with OLZ leads to a decrease in dopamine levels. Intriguingly, the administration of probiotics, either alone or in combination with OLZ, resulted in higher dopamine levels than those observed in rats treated solely with OLZ. These results suggest that probiotics may have the potential to mitigate the dopaminergic suppression associated with OLZ treatment, highlighting a promising avenue for enhancing dopaminergic activity and possibly counteracting some of the adverse effects of OLZ on the dopaminergic system. Probiotics can also help counteract the metabolic side effects of OLZ by indirectly influencing glucose metabolism through the γ-aminobutyric acid pathway. Furthermore, probiotics have been shown to enhance the incretin effect, improving insulin secretion and sensitivity through glucagon-like peptide-1 (GLP-1) receptor pathways [53]. Further research is needed to fully understand the role of GLP-1 in mediating the effects of probiotics on OLZ-induced metabolic changes.

The H&E-stained tissue results highlighted the differential impacts of low and high doses of probiotic administration and OLZ treatment on colon and liver health in the experimental groups. These findings can be contextualised within the broader scientific dialogue regarding the gut-liver axis, the role of probiotics in mitigating drug-induced side effects, and the pathophysiology of MS. OLZ disrupts metabolic homeostasis, leading to features such as fatty liver (steatosis) and intestinal damage [33]. The observed steatosis and intestinal crypt damage in the OLZ-treated group underscore these adverse effects.

The findings that both low and high doses of probiotics maintained the normal colon epithelium and prevented OLZ-induced intestinal damage are consistent with studies suggesting that probiotics can restore gut barrier integrity and reduce inflammation [54]. Probiotics have been shown to exert protective effects against various gastrointestinal disturbances, including those induced by medications [55].

The differential impacts of probiotic doses on liver architecture, with high doses administered along with OLZ showing a protective effect against OLZ-induced steatosis, mirror the emerging evidence that probiotics can ameliorate drug-induced liver injury [56]. This protective effect is possibly mediated through the modulation of the GM, a reduction in intestinal permeability, and a subsequent decrease in endotoxin levels in the liver, thus reducing inflammatory and fibrotic processes [57].

Probiotics may protect against metabolic disturbances through several mechanisms, including modulating the GM composition, enhancing intestinal barrier function, and regulating inflammatory responses. These effects collectively reduce metabolic endotoxemia, a condition associated with MS and nonalcoholic fatty liver disease [58]. This brief synthesis contextualises the experimental findings within the existing body of research, underscoring the potential of probiotics as a therapeutic strategy to mitigate some of the adverse effects of OLZ, particularly concerning MS and liver health.

## Directions for future research

SCFA quantification and immunohistochemistry of brain tissue could have provided additional insights into the potential mechanism by which gut-brain axis modulation prevents MS.

In the subsequent phase of our investigation, we plan to delve into the antipsychotic effects of the probiotics, during which we will examine both central 5-HT and dopamine levels. Given that our initial focus was primarily on parameters related to metabolic syndrome, we limited our analysis to peripheral 5-HT and dopamine levels.

## Limitations

- Since a combination of probiotics was utilised, the specific microbe reducing the symptoms of OLZ-induced MS could not be identified.
- Although this study suggested a probiotic effect on the GM, it did not fully elucidate the mechanisms underlying these changes or their direct link to MS.

## Conclusion

This study concluded that administering OLZ for 3 months leads to significant metabolic side effects, including weight gain, elevated lipid profiles, increased blood pressure, and altered GM. However, probiotic treatment shows promise in mitigating these adverse effects by modulating the GM, suggesting a beneficial role in managing OLZ-induced MS.

## Supplementary Information

Supplementary Material 1.

## Data Availability

No datasets were generated or analysed during the current study.

## Abbreviations

ANOVA: Analysis of variance
BMI: Body mass index
BW: Body weight
CNS: Central nervous system
CV: Central vein
DNA: Deoxyribose nucleic acid
ELISA: Enzyme-linked immunosorbent assay
GM: Gut microbiota
H&E: Hematoxylin and eosin
HDL-C: High-density lipoprotein cholesterol
IAEC: Institutional Animal Ethics Committee
MAP: Mean arterial pressure
MDA: Mean decrease accuracy
MDA: Mean decrease in accuracy
MGBA: Microbiota–gut–brain axis
MS: Metabolic syndrome
N: Normal control
NIBP: Noninvasive blood pressure
ocuff: Occlusion cuff
OLZ: Olanzapine
PCR: Polymerase chain reaction
PH: High-dose probiotic
PM: Probiotic low dose
RA: Relative abundance
RF: Random forest
rRNA: Ribosomal ribonucleic acid
SCFAs: Short-chain fatty acids
SEM: Standard error of the mean
SPSS: Statistical Package for the Social Sciences
TC: Total cholesterol
TG: Triglyceride
TH: Olanzapine + high-dose probiotics
TM: Olanzapine + low-dose probiotics
*TM7*: *Saccharibacteria*
TNF-α: Tumor necrosis factor-α
VPR: Volume pressure recording
