# Supplementary material for: Exploring the effects of probiotics on olanzapine-induced metabolic syndrome through the gut microbiota
Source: Gut Pathog. 2024 Dec 21;16:77. doi: 10.1186/s13099-024-00664-2 (PMC11662719; doi:10.1186/s13099-024-00664-2)
Supplement: Supplementary file 1 — Supplementary Material 1. [file 13099_2024_664_MOESM1_ESM.docx]

**2.1** **Animal preparation for blood pressure measurement**

**2.1.1 Setup:** Animals were placed in holders with their tails used for positioning, ensuring no pinching of body parts. The setup included adjusting the nose cone and securing the holder’s rear hatch (**Figure 1-C**). Holders were then positioned on a warming platform, with positions rotated for consistent BP measurements across sessions (**Figure 1-B**). A 5-minute acclimation period was provided.

**2.1.2 Cuff Placement**: The occlusion cuff (ocuff) was placed near the tail's base, with a secure attachment to the holder (**Figure 1-C, F**), followed by the placement of the volume pressure recording (VPR) Cuff close to the ocuff and secured on the holder (**Figure 2**). Connections were made to the CODA monitor (**Figure 1-D**).

**2.1.3 Temperature Regulation**: Animals were allowed 5 minutes for thermoregulation, with temperatures between 32 to 35°C, monitored by an infrared thermometer (**Figure 1-E**) and regulated with a warming cover (**Figure 1-A**). Pre-study acclimation involved 15-minute daily holder sessions for a week (**Figure 1-C**). Blood pressure was recorded thrice per session to derive mean arterial pressure (MAP) for analysis, with measurements taken at baseline and every 15 days over 3 months.


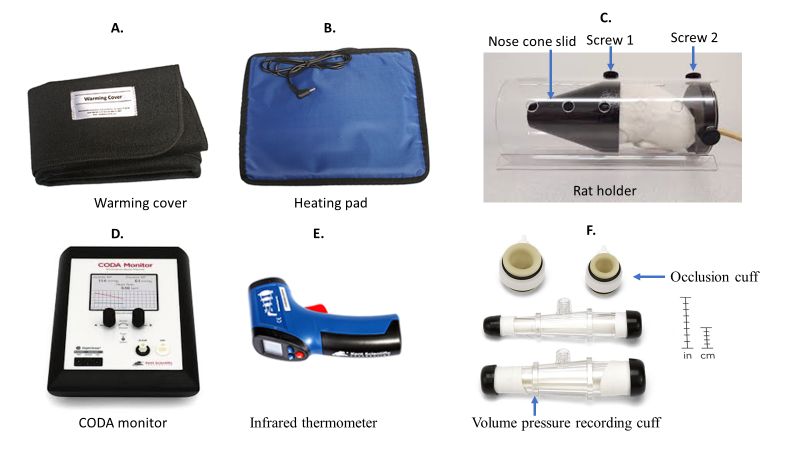


**Suppl. Figure 1: Different parts of the CODA NIBP instrument.**


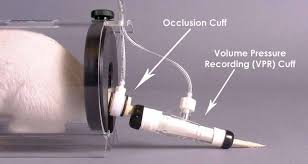


**Suppl. Figure 2: Rat holder with VPR cuff and ocuff of CODA NIBP instrument.**

Å- as compared to (vs) O (p<0.001); μ - vs NC (p<0.001); κ - vs probiotic-I (p<0.001); λ- vs probiotic-II (p<0.001); δ – vs probiotic (I and II) and test groups (I and II) (p<0.00; γ- vs NC, O, Test-I, Test-II (p<0.001).

$ €

* $ €

**Suppl. Figure 3: Mean of bodyweight in grams comparison** **between various experimental groups at baseline at the end of 90 days.**

Å - As compared to (vs) O (p<0.001); μ – vs N (p<0.001); δ – vs probiotic (I and II) and test groups (I and II) (p<0.001); * - vs O (p<0.05); $ - vs probiotic-I (p<0.05); £ - vs Test-I (p<0.05).

**Suppl. Figure 4: TC levels in mg/dL comparison at baseline and the end of 90 days of treatment in various treatment groups.**

Å - As compared to (vs) O (p<0.001); μ – vs N (p<0.001); δ – vs probiotic (I and II) and test groups (I and II) (p<0.001).

**Suppl. Figure 5: TG levels in mg/dL comparison at baseline and the end of 90 days of treatment in various treatment groups.**

* - As compared to (vs) OLZ (p<0.05); κ – vs probiotic-I (p<0.001); λ - vs probiotic-II (p<0.001); # - vs N (p<0.05); £ - vs Test-I (p<0.05), γ - vs N, OLZ, Test-I, Test-II (p<0.001).

**Suppl. Figure 6: HDL-C levels in mg/dL comparison at baseline and the end of 90 days of treatment in various treatment groups.**

* - As compared to (vs) O (p<0.05); β – vs probiotic (I and II) and test groups (I and II) (p<0.05); # - vs N (p<0.05).

**Suppl. Figure 7: Serum serotonin levels in ng/ml at baseline and the end of 90 days of treatment in various treatment groups.**

* - As compared to (vs) O (p<0.05); β – vs probiotic (I and II) and test groups (I and II) (p<0.05); # - vs NC (p<0.05)

**Suppl. Figure 8: Serum dopamine levels in ng/ml at baseline and the end of 90 days of treatment in various treatment groups.**

**
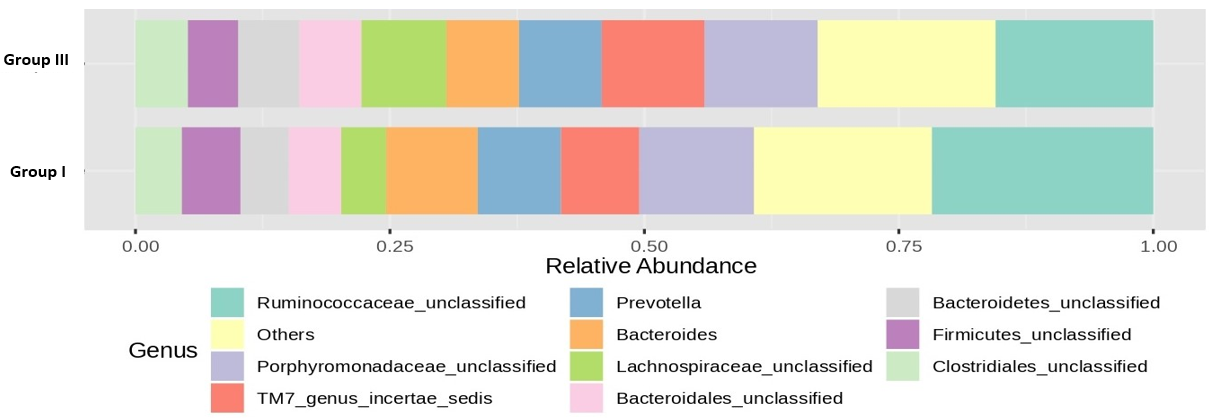
**

**Suppl. Figure 9: Bar plots of groups I and III RA at the genera level after 90 days of treatment.**

**
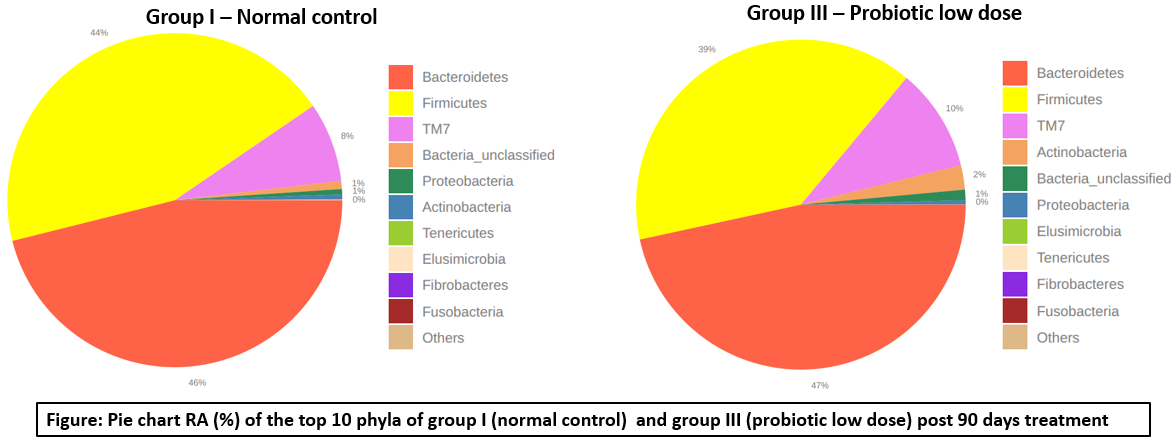
**

**Suppl. Figure 10: Pie chart of RA (%) of the top 10 phyla of groups I and III post 90 days treatment.**

**
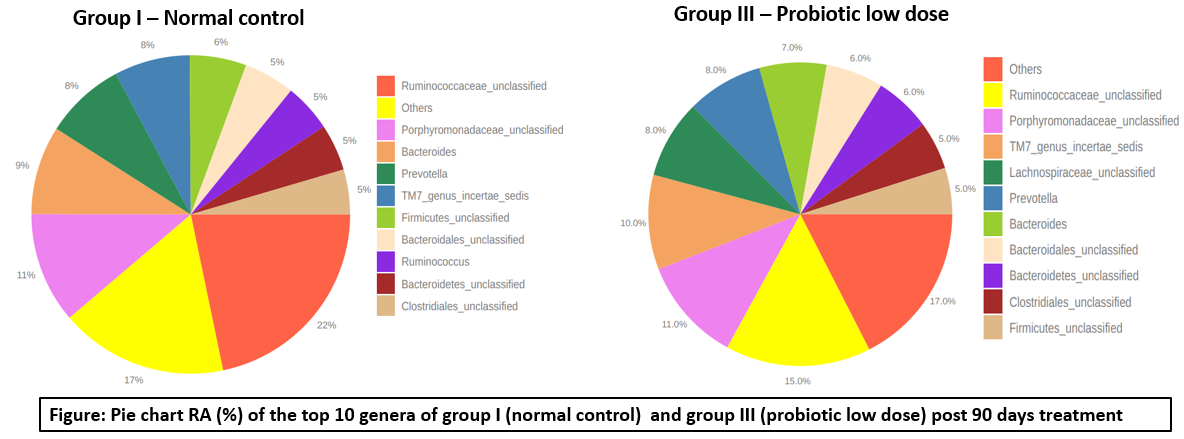
**

**Suppl. Figure 11: Pie chart of RA (%) of the top 10 genera of groups I and III post 90 days of treatment.**

**
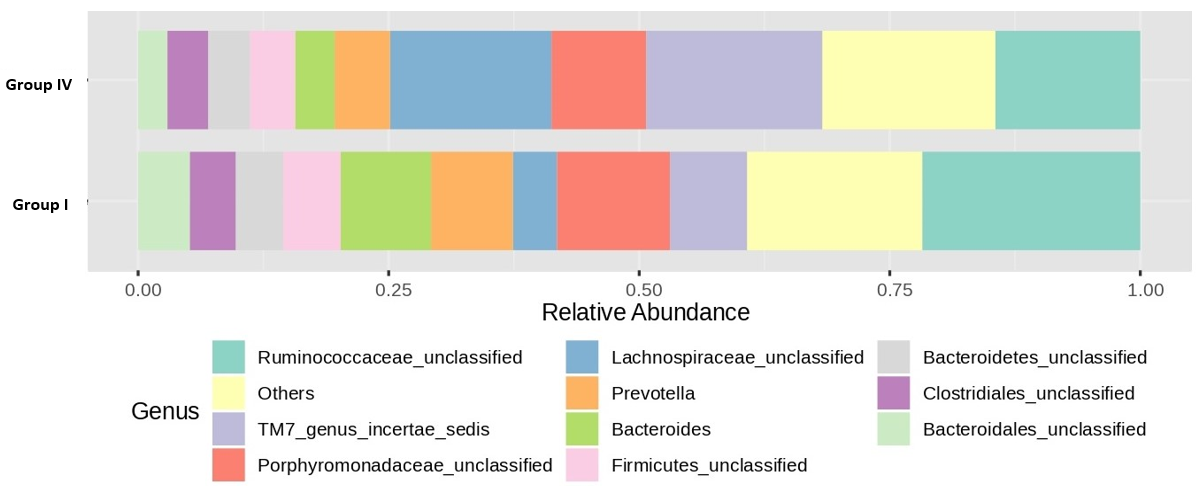
**

**Suppl. Figure 12: Bar plots of groups I and IV RA at the genus level after 90 days of treatment.**

**
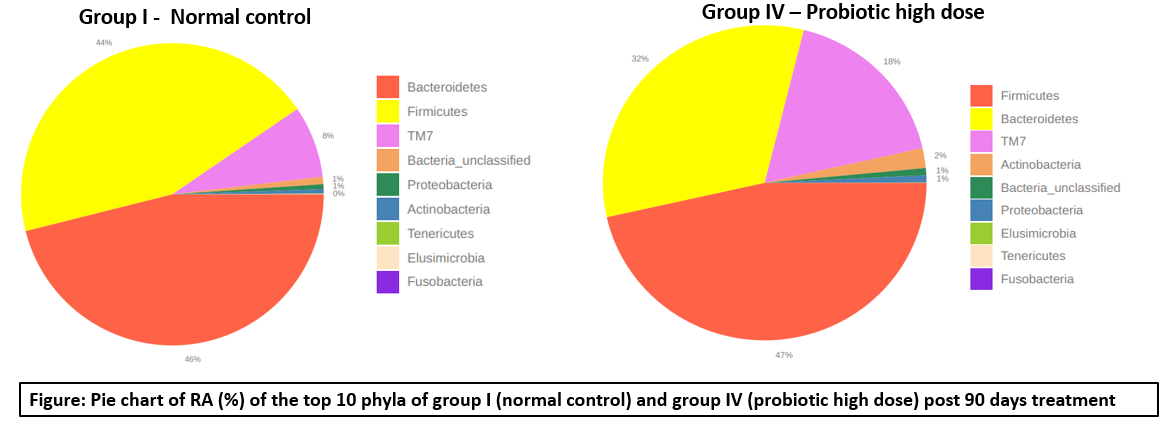
**

**Suppl. Figure 13: Pie chart of RA (%) of the top 10 phyla of groups I and IV post 90 days treatment.**

**
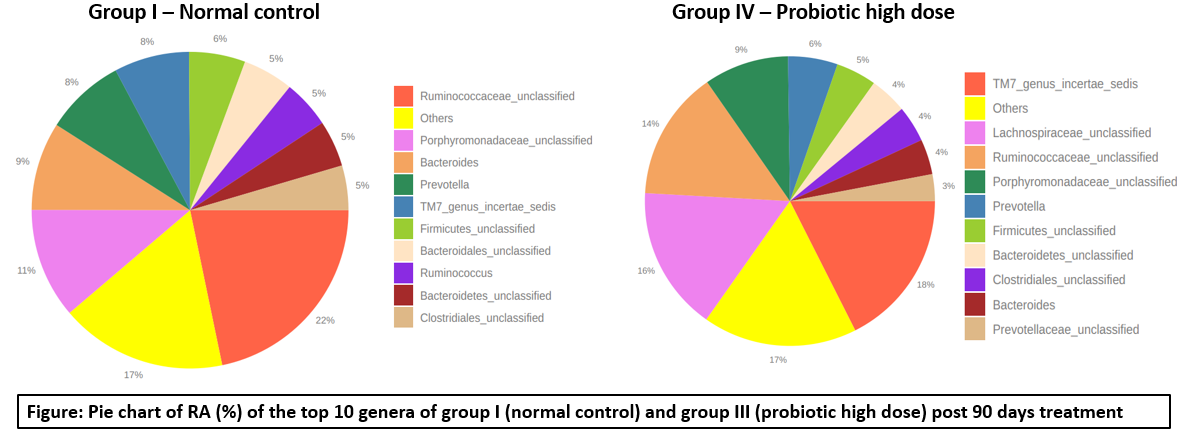
**

**Suppl. Figure 14: Pie chart of RA (%) of the top 10 genera of groups I and IV post 90 days of treatment.**

**
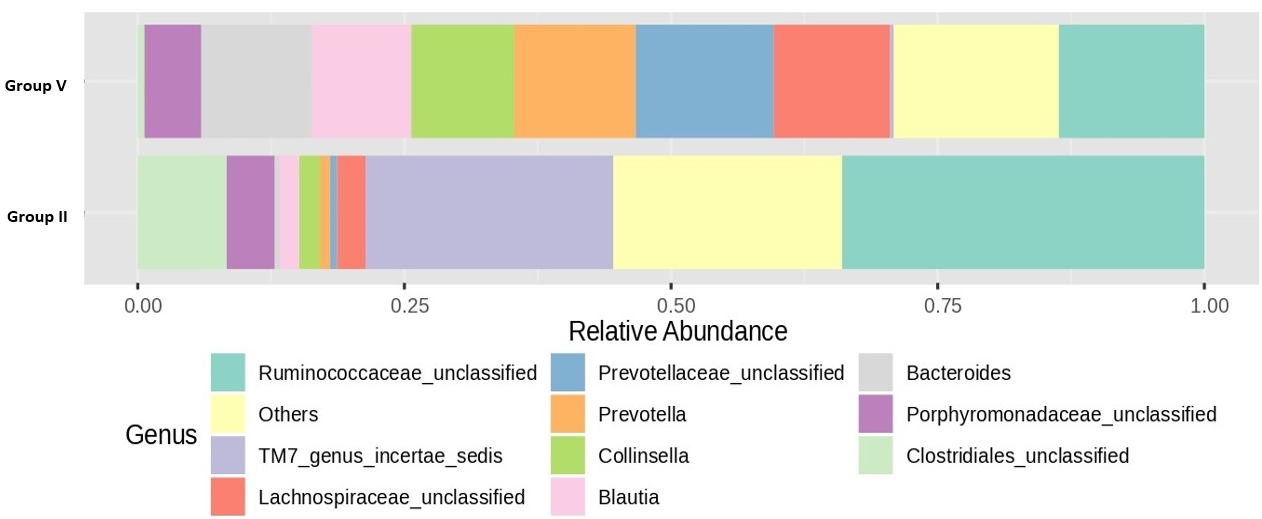
**

**Suppl. Figure 15: Bar plots of groups II and V RA at the genus level after 90 days of treatment.**


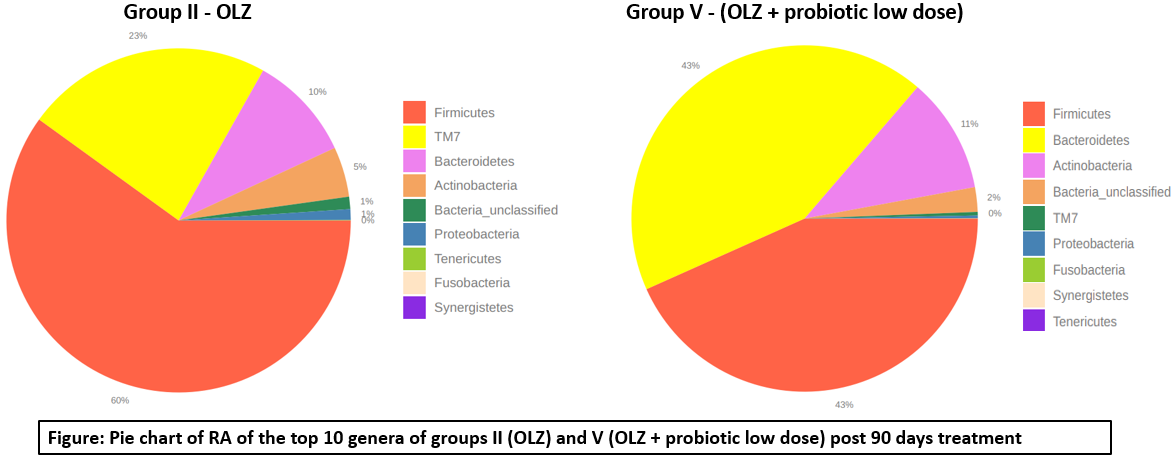


**Suppl. Figure 16: Pie chart of RA (%) of the top 10 phyla of groups II and V post 90 days treatment.**


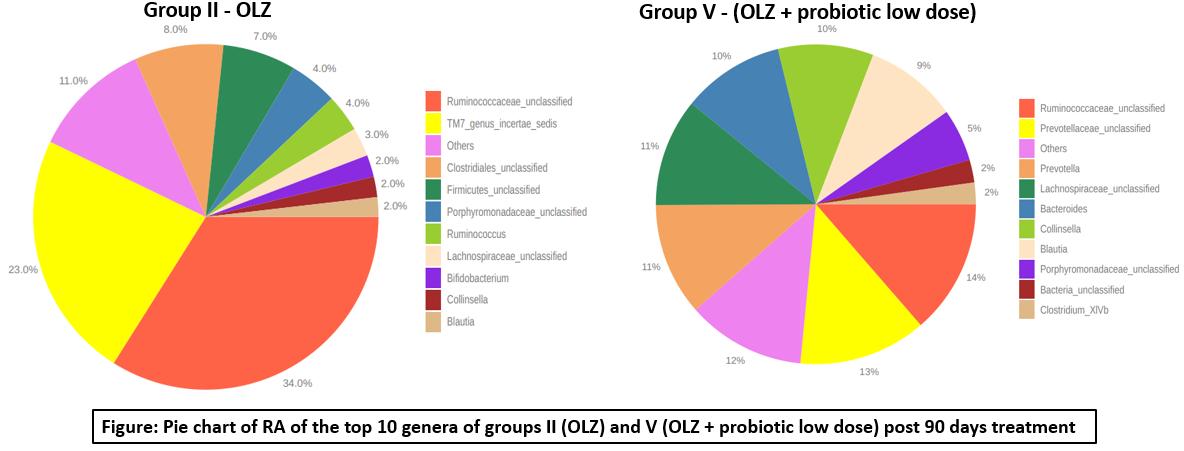


**Suppl. Figure 17: Pie chart of RA (%) of the top 10 genera of groups II and V post 90 days of treatment.**

**Suppl. Table 1: Comparison of BW(g) between various experimental groups**

| **Group (n=6)** | **BW - baseline** | **BW - 15 days** | **BW - 30 days** | **BW - 45 days** | **BW - 60 days** | **BW - 75 days** | **BW - 90 days** |  |
| --- | --- | --- | --- | --- | --- | --- | --- | --- |
| Group I: Normal control (N) | 250.67 ± 0.67 | 254.34 ± 1.09 | 259.67 ± 1.12 | 262.67 ± 1.12 | 267.5 ± 1.31 | 272.33 ± 1.2 | 274.83 ± 1.14 |  |
| Group II: OLZ (O) | 250.34 ± 0.67 | 271 ± 0.86 **μκλδ** | 265 ± 1.84 **δ** | 275.83 ± 3.84 **#δ** | 291.83 ± 2.59 **μδ** | 302.50 ± 1.65 **μδ** | 312 ± 1.15 **μδ** |  |
| Group III: Probiotic I (probiotic low dose) - PM | 251 ± 0.82 | 242.67 ± 2.92 **#Å£&** | 244 ± 2.57 **Åμδ** | 246.33 ± 2.38 **Åμ£&** | 249.33 ± 2.09 **γ** | 251 ± 1.91 **γ** | 257 ± 1.59 **γ** |  |
| Group IV: Probiotic II (probiotic high dose) - PH | 249.83 ± 0.87 | 242.17 ± 1.19 **Åμ£&** | 245.83 ± 1.51  **Åμ£&** | 247 ± 1.81 **Åμ£&** | 246 ± 2.02 **γ** | 247 ± 1.90 **γ** | 251.67 ± 1.58 **γ** |  |
| Group V: OLZ + probiotic- I (TM) | 248.33 ± 0.88 | 251 ± 2.03 **Å£&** | 254.83 ± 0.75 **Åκ@** | 258.83 ± 1.08 **Å$@** | 264.17 ± 1.30 **Åκλ** | 265.17 ± 1.70 **Åκλ** | 269 ± 1.24 **Åκλ** |  |
| Group VI: OLZ + probiotic- II (TH) | 249.5 ± 0.99 | 251.5 ± 1.15 **Å£&** | 254.17 ± 0.87 **Åκ@** | 258.17 ± 0.60 **Å$@** | 263.83 ± 0.79 **Åκλ** | 268.17 ± 0.48 **Åκλ** | 276.67 ± 0.71 **Åκλ** |  |
| *- Compared to (vs) O (p<0.05); Å- vs O (p<0.001); μ - vs N (p<0.001); κ - vs PM (p<0.001); λ- vs PH (p<0.001); δ – vs groups PM, PH, TM, and TH (p<0.001), #- vs N (p<0.05); $ - vs PM (p<0.05); @ - vs PH (p<0.05); £- vs TM (p<0.05), & - vs. TH (p<0.05); γ- vs. N, O, TM, TH (p<0.001).  Values are represented as the mean ± SEM, n - number of rats in each group; SEM - Standard Error of Mean, BW- body weight in grams. | | | | | | | | |

**Suppl. Table 2: Comparison of MAP (mm of Hg) levels** **among the various experimental groups**

| **Group (n=6)** | **MAP - baseline** | **MAP- 15 days** | **MAP- 30 days** | **MAP- 45 days** | **MAP - 60 days** | **MAP- 75 days** | **MAP- 90 days** |
| --- | --- | --- | --- | --- | --- | --- | --- |
| Group I: Normal control (N) | 83.22 ± 0.99 | 84 ± 1.92 | 87.45 ± 1.60 | 84.22 ± 1.82 | 84.72 ± 1.85 | 86.73 ± 0.82 | 87.82 ± 1.26 |
| Group II: OLZ (O) | 83.34 ± 1.11 | 107.67 ± 6.42 **μκλ** | 100.28 ± 3.60 **$λ£** | 112.95 ± 4.83 **μδ** | 125.44 ± 2.54 **μδ** | 141.08 ± 2.80 **μδ** | 144.3 ± 2.66 **μδ** |
| Group III: Probiotic I (probiotic low dose) - PM | 83.94 ± 1.40 | 88 ± 1.64 **Å** | 85.33 ± 1.41 ***** | 71.22 ± 1.55 **Å** | 84.44 ± 1.58 **Å** | 93.5 ± 2.04 **Å£** | 85.27 ± 1.54 **Å** |
| Group IV: Probiotic II (probiotic high dose) - PH | 85.11 ± 1.38 | 81.94 ± 4.92 **Å** | 73.72 ± 3.47 ***** | 75.83 ± 2.30 & | 88.67 ± 2.40 **Å** | 90.60 ± 2.16 **Å** | 88.25 ± 3.11 **Å** |
| Group V: OLZ + probiotic- I (TM) | 84.64 ± 1.11 | 94.42 ± 2.33 * | 84.53 ± 3.46 ***** | 83.26 ± 3.66 **Å** | 78.85 ± 3.50 **Å&** | 82.76 ± 2.53 **Å$&** | 90.57 ± 2.81 **Å** |
| Group VI: OLZ + probiotic- II (TH) | 85.56 ± 0.89 | 102.82 ± 3.39 **#** | 95.99 ± 3.81**@** | 86.87 ± 4.42 **Å$** | 98.54 ± 5.22 **Å£** | 93.24 ± 2.45 **Å£** | 92.81 ± 2.31 **Å** |
| *- Compared to (vs) O (p<0.05); Å- vs O (p<0.001); μ – vs N (p<0.001); κ – vs PM (p<0.001); λ- vs PH (p<0.001); δ – vs groups PM, PH, TM, and TH (p<0.001), #- vs N (p<0.05); $ - vs PM (p<0.05); @ - vs PH (p<0.05); £- vs TM (p<0.05), & - vs. TH (p<0.05); γ- vs. N, O, TM, TH (p<0.001).  Values are represented as the mean ± SEM, n - number of rats in each group; SEM - Standard Error of Mean, and MAP values are expressed in mm of Hg. | | | | | | | |

**Suppl. Table 3: Serum serotonin and dopamine levels in ng/ml at baseline and the end of 90 days**

| **Group (n=6)** | **Serotonin- Baseline** | **Serotonin after 90 days** | **Dopamine - Baseline** | **Dopamine after 90 days** |
| --- | --- | --- | --- | --- |
| Group I: Normal control (N) | 17.3 ± 0.23 | 17.37 ± 0.23 | 1.74 ± 0.03 | 1.75 ± 0.02 |
| Group II: OLZ (O) | 17.3 ± 0.37 | 15 ± 0.72 **#β** | 1.73 ± 0.03 | 1.45 ± 0.09 **#β** |
| Group III: Probiotic I (probiotic low dose) - PM | 17.22 ± 0.27 | 18.45 ± 0.42 ***** | 1.74 ± 0.02 | 1.82 ± 0.02 ***** |
| Group IV: Probiotic II (probiotic high dose) - PH | 17.37 ± 0.25 | 18.32 ± 0.18 ***** | 1.73 ± 0.03 | 1.77 ± 0.03 ***** |
| Group V: OLZ + probiotic- I (TM) | 17.2 ± 0.29 | 18.1± 0.24 ***** | 1.73 ± 0.02 | 1.79 ± 0.03 ***** |
| Group VI: OLZ + probiotic- II (TH) | 17.21 ± 0.25 | 17.87 ± 0.17***** | 1.73 ± 0.02 | 1.79± 0.03 ***** |
| * - Compared to (vs) OLZ (p<0.05); β – vs groups (III, IV, V, and VI) (p<0.05); # - vs N (p<0.05).  Values are represented as the mean ± SEM, n - number of rats in each group; SEM - Standard Error of Mean, serotonin levels are expressed in ng/ml. | | | | |
